# Synthesis and _In Vitro_ Antimycobacterial Activity of Novel *N*-Arylpiperazines Containing an Ethane-1,2-diyl Connecting Chain

**DOI:** 10.3390/molecules22122100

**Published:** 2017-11-30

**Authors:** Tomáš Goněc, Ivan Malík, Jozef Csöllei, Josef Jampílek, Jiřina Stolaříková, Ivan Solovič, Peter Mikuš, Stanislava Keltošová, Peter Kollár, Jim O’Mahony, Aidan Coffey

**Affiliations:** 1 Department of Chemical Drugs, Faculty of Pharmacy, University of Veterinary and Pharmaceutical Sciences in Brno, Palackého 1946/1, Brno CZ-612 42, Czech Republic; gonect@vfu.cz (T.G.); csolleij@vfu.cz (J.Cs.); 2 Department of Pharmaceutical Chemistry, Faculty of Pharmacy, Comenius University in Bratislava, Odbojárov 10, Bratislava SK-832 32, Slovak Republic; malikivan001@gmail.com (I.M.); josef.jampilek@gmail.com (J.J.); 3 Laboratory for Mycobacterial Diagnostics and Tuberculosis, Regional Institute of Public Health, Partyzánské náměstí 7, Ostrava CZ-702 00, Czech Republic; Jirina.Stolarikova@zu.cz (J.S.); 4 Clinic for Tuberculosis and Lung Diseases, National Institute for Tuberculosis, Lung Diseases and Thoracic Surgery, Vyšné Hágy, Vysoké Tatry SK-059 84, Slovak Republic; solovic@hagy.sk (I.S.); 5 Department of Public Health, Faculty of Health, Catholic University in Ružomberok, Hrabovská cesta 1A, Ružomberok SK-034 01, Slovak Republic; ivan.solovic@ku.sk (I.S.); 6 Department of Pharmaceutical Analysis and Nuclear Pharmacy, Faculty of Pharmacy, Comenius University in Bratislava, Odbojárov 10, Bratislava SK-832 32, Slovak Republic; mikus@fpharm.uniba.sk (P.M.); 7 Department of Human Pharmacology and Toxicology, University of Veterinary and Pharmaceutical Sciences in Brno, Palackého 1946/1, Brno CZ-612 42, Czech Republic; kollarp@vfu.cz (P.K.); keltosovas@vfu.cz (S.K.); 8 Department of Biological Sciences, Cork Institute of Technology, Bishopstown, Cork T12 P928, Ireland; jim.omahony@cit.ie (J.O.M.); aidan.coffey@cit.ie (A.C.)

**Keywords:** *N*-arylpiperazines, arylaminoethanols, lipophilicity, electronic properties, *Mycobacterium tuberculosis* H_37_R_v_

## Abstract

Novel 1-(2-{3-/4-[(alkoxycarbonyl)amino]phenyl}-2-hydroxyethyl)-4-(2-fluorophenyl)-piperazin-1-ium chlorides (alkoxy = methoxy to butoxy; **8a**–**h**) have been designed and synthesized through multistep reactions as a part of on-going research programme focused on finding new antimycobacterials. Lipophilic properties of these compounds were estimated by RP-HPLC using methanol/water mobile phases with a various volume fraction of the organic modifier. The log *k*_w_ values, which were extrapolated from intercepts of a linear relationship between the logarithm of a retention factor *k* (log *k*) and volume fraction of a mobile phase modifier (*ϕ*_M_), varied from 2.113 (**8e**) to 2.930 (**8h**) and indicated relatively high lipophilicity of these salts. Electronic properties of the molecules **8a**–**h** were investigated by evaluation of their UV/Vis spectra. In a next phase of the research, the compounds **8a**–**h** were _in vitro_ screened against *M. tuberculosis* CNCTC My 331/88 (identical with H_37_R_v_ and ATCC 2794), *M. kansasii* CNCTC My 235/80 (identical with ATCC 12478), a *M. kansasii* 6 509/96 clinical isolate, *M. avium* CNCTC My 330/80 (identical with ATCC 25291) and *M. avium intracellulare* ATCC 13950, respectively, as well as against *M. kansasii* CIT11/06, *M. avium* subsp. *paratuberculosis* CIT03 and *M. avium hominissuis* CIT10/08 clinical isolates using isoniazid, ethambutol, ofloxacin, ciprofloxacin or pyrazinamide as reference drugs. The tested compounds **8a**–**h** were found to be the most promising against *M. tuberculosis*; a *MIC* = 8 μM was observed for the most effective 1-(2-{4-[(butoxycarbonyl)amino]phenyl}-2-hydroxyethyl)-4-(2-fluorophenyl)piperazin-1-ium chloride (**8h**). In addition, all of them showed low (insignificant) _in vitro_ toxicity against a human monocytic leukemia THP-1 cell line, as observed *LD*_50_ values > 30 μM indicated. The structure–antimycobacterial activity relationships of the analyzed **8a**–**h** series are also discussed.

## 1. Introduction

**Figure 1:**
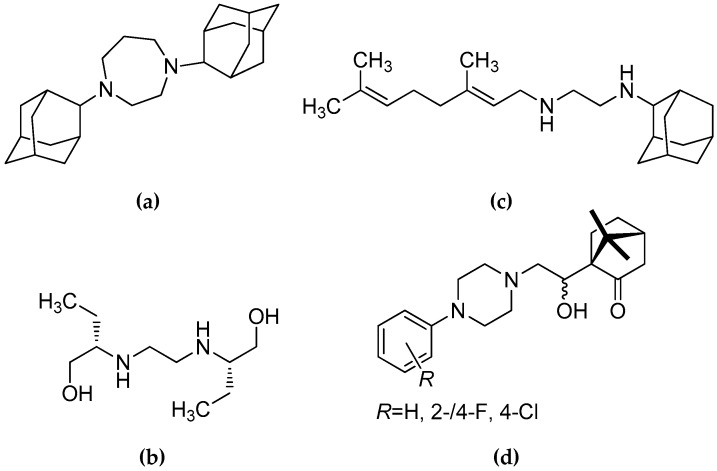
Chemical structures of: (**a**) compound **SQ775**; (**b**) ethambutol (EMB); (**c**) *N*-geranyl-*N*´-(2-adamanthan-1-yl)ethane-1,2-diamine; and (**d**) chiral *N*-arylpiperazine-based aminoalcohols (***N*-ArPA**), which showed a notable _in vitro_ efficiency against *M. tuberculosis* CNCTC My 331/88 [9,10,11,12,13].

An *N*-arylpiperazine privileged scaffold [1] has been found in the chemical structure of many effective antimycobacterial agents [2,3,4,5,6,7,8]. Some of these compounds have been characterized by a very typical structural arrangement [5,6,7,8], i.e., the *N*-aryl- or variously substituted *N*-phenylpiperazine moiety, connecting hydrocarbon chain and terminal heterocyclic fragment. Efforts to combine the *N*-arylpiperazine and “ethambutol-like“ structural frameworks were supported by comprehensive structure–activity relationships (*SAR*) studies of homopiperazin-1,4-diyl-containing derivatives (e.g., a molecule **SQ775**; Figure 1a), ethambutol (EMB; Figure 1b) and the diamines structurally based on EMB [9,10,11,12]. Among the synthesized compounds, *N*-geranyl-*N*′-(2-adamanthan-1-yl)ethane-1,2-diamine (Figure 1c) showed promising efficiency [12]. The outlined systematic research led to ***N*-ArPA** molecules (Figure 1d), in which structure the distance between two nitrogens, presence of *β*-aminoalcohol motifs and short connecting chains were crucial for their _in vitro_ antimycobacterial activity [13]. The derivatives ***N*-ArPA** were very effective against *Mycobacterium tuberculosis* CNCTC My 331/88 (identical with *M. tuberculosis* H_37_R_v_) and multi-drug resistant (MDR) *M. tuberculosis* 43 strain, which showed resistance to rifampicin (RIF) and isoniazid (INH).

It was also concluded that removal or significant alteration of basicity of either amino group led to loss of potency [9,13]. In addition, the presence of the *R* = 2-/4-F substituent and OH group with the (*R*)-configuration at the carbon of a connecting chain (Figure 1d) resulted in higher _in vitro_ antimycobacterial efficiency than in a case of EMB. On the other hand, the remaining *R* substituents (H, Cl; Figure 1d) caused decrease in activity [13].

Regarding the design of original antimycobacterials, a carbamate (NHCOO) functionality is structurally related to hybrid amide-ester features and, in general, displays very good chemical and proteolytic stabilities [14]. The carbamate functionality imposes a degree of conformational restriction due to the delocalization of non-bonded electrons on nitrogen into the carboxyl moiety. In addition, this functionality participates in hydrogen bonding through the carboxyl group and the backbone NH [15]. Therefore, a substitution on the *O*- and *N*-termini of the carbamate offers opportunities to modulate biological properties and improve stability and pharmacokinetic features [14,15].

The idea to introduce a lipophilic 3-/4-alkoxy or 3-/4-alkoxycarbonylamino moiety (alkoxy = methoxy to butoxy) into a chemical structure of newly designed molecules was based on previous studies focused on the synthesis and _in vitro_ biological evaluation of the *N*-arylpiperazines and phenylcarbamic acid derivatives [16,17,18,19,20,21]. Their antimycobacterial activity increased with elongation of this chain until a maximum in efficiency was reached [19,20,21]. Further increase in its length led to the decrease in potency. The observed dependence was approximated by a parabolic function and described as a cut-off effect. That phenomenon was comprehensively reviewed and rationalized in number of mechanistic ways by Hansch and Clayton [22] as well as Balgavý and Devínsky [23] more than two decades later.

Regarding the conclusions published in papers [19,20,21], it might be expected that eventual incorporation of the alkoxycarbonylamino group into a 2-position of a phenyl ring would cause the decrease in antimycobacterial activity.

It was also believed that the presence of the linear alkoxy side chain would be very favorable in terms of interactions with target structures located in biomembranes of mycobacterial strains, especially *M. tuberculosis* H_37_R_v_. Branching or substitution of the alkoxy with the alkyl group is reported to have caused decrease in efficiency [19,20,21,24].

**Figure 2:**
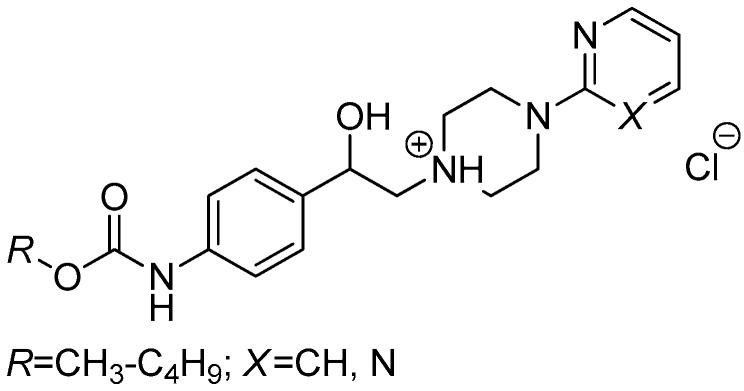
*N*-Arylpiperazine derivatives containing a 2-hydroxyethane-1,2-diyl connecting chain (a series **MM**), which were _in vitro_ screened against some mycobacterial strains [18].

It was found that a suitable modification of the aromatic ring attached to a piperazin-1,4-diyl framework might not result in loss of activity. The derivatives containing a pyrimidin-2-yl fragment were slightly more efficient than the ones with a pyridin-2-yl moiety (a series **MM**; Figure 2). The molecules **MM** [18] were able to effectively _in vitro_ fight *M. tuberculosis* My 331/88, *M. kansasii* 6 509/96, *M. tuberculosis* 7375/1998, a strain resistant to INH, RIF, rifabutine (RFB) and streptomycin (STM), respectively, as well as *M. tuberculosis* Prague 1, an extremely-resistant strain to INH, RIF, RFB, STM, EMB, ofloxacin (OFLX), gentamicin (GTM) and amikacin (AK), respectively [18].

Inspired and encouraged by given importance of the 3-/4-alkoxycarbonylamino, *β*-aminoalcohol and 4-(2-fluorophenyl)piperazin-1-yl fragments in a chemical structure of effective antimycobacterials, the present study was focused on the synthesis of original racemic compounds in order to find out if they would be efficient against some of following mycobacterial strains _in vitro_, namely, *M. tuberculosis* CNCTC My 331/88 (identical with H_37_R_v_ and ATCC 2794), *M. kansasii* CNCTC My 235/80 (identical with ATCC 12478), a *M. kansasii* 6 509/96 clinical isolate, *M. avium* CNCTC My 330/80 (identical with ATCC 25291) as well as *M. avium intracellulare* ATCC 13950, *M. kansasii* CIT11/06, *M. avium* subsp. *paratuberculosis* CIT03 and *M. avium hominissuis* CIT10/08 clinical isolates, respectively.

Despite a fact that currently designed molecules contained a stereogenic centre (Table 1), the synthesis of racemates has been regarded as a reasonable strategy in conceptual development of original antimycobacterial agents to verify the relevancy of proposed structural frameworks [16,18,25,26,27].

## 2. Results and Discussion

### 2.1. Chemistry

#### 2.1.1. Synthesis and Spectral Characteristics

Designer *N*-arylpiperazines were synthesized *via* multistep reactions, exploring the impact of their lipohydrophilic and electronic properties on the _in vitro_ activity against selected mycobacterial strains. In addition, the _in vitro_ toxicity profile of the final molecules against a human monocytic leukemia THP-1 cell line was inspected.

**Scheme 1:**
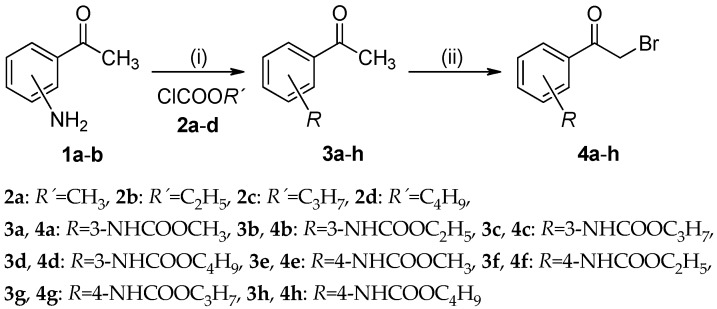
Synthesis of the alkyl [3-/4-(bromoacetyl)phenyl]carbamates 4a–h (alkyl = methyl to butyl), *Reagents and conditions*: (i) ClCOO*R*′ (*R*′ = methyl to butyl; 2a–d), pyridine; (ii) Br_2_, chloroform.

**Scheme 2:**
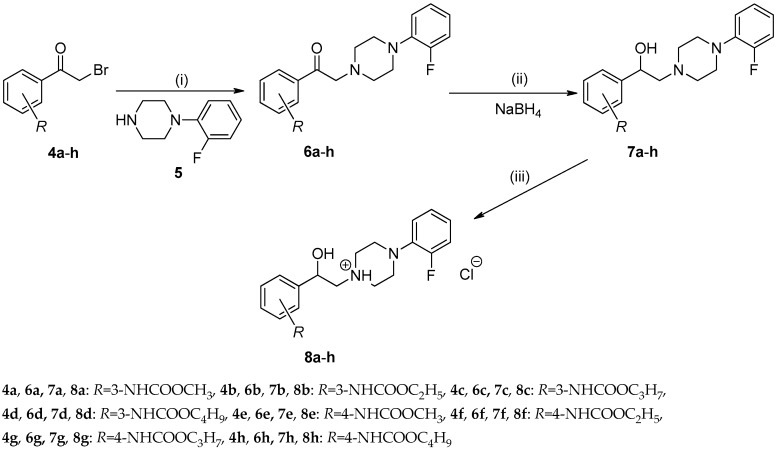
Synthesis of the final 1-(2-{3-/4-[(alkoxycarbonyl)amino]phenyl}-2-hydroxyethyl)-4-(2-fluorophenyl)piperazin-1-ium chlorides **8a**–**h** (alkoxy = methoxy to butoxy), *Reagents and conditions*: (i) TEA, THF; (ii) NaBH_4_, methanol; (iii) a saturated solution of hydrogen chloride in diethyl ether.

Following the outlined objectives, the compounds were synthesized according to Scheme 1 and Scheme 2 as follows. Initially, 3-aminoacetophenone (**1a**) and 4-aminoacetophenone (**1b**) were employed as convenient starting compounds. They were treated with alkyl chloroformates **2a**–**d** (alkyl = methyl to butyl) at presence of pyridine in acetone to afford colourless alkyl (3-/4-acetylphenyl)-carbamates **3a**–**h** [28] in the yields that varied from 89% to 99% (Scheme 1).

When designing the chemical structure of target molecules, 2-aminoacetophenone was not considered a suitable starting structure due to a possible undesired cyclization of resulting intermediates [29,30]. In addition, a weak _in vitro_ antimycobacterial activity of final products would be probably observed [19,20,21].

The molecules **3a**–**h** underwent *α*-bromination of an acetyl group because of the dropwise addition bromine in chloroform. This reaction procedure took place at a sufficiently high rate in chloroform at room temperature with constant stirring [31]. Resulting alkyl [3-/4-(bromoacetyl)phenyl]carbamates **4a**–**h** (alkyl = methyl to butyl; Scheme 1) were achieved with 75% to 93% yields.

A substitution of bromine by 1-(2-fluorophenyl)piperazine **5** [32] in the presence of triethylamine (TEA) in anhydrous tetrahydrofuran (THF) led to colourless alkyl {3-/4-[(4-(2-fluorophenyl)piperazin-1-yl)acetyl]phenyl}carbamates **6a**–**h** (Scheme 2). The intermediates **6a**–**h** were prepared in moderate to good yields that ranged from 75% to 96%. Next, they were transformed into alkyl {3-/4-[2-(4-(2-fluorophenyl)piperazin-1-yl)-1-hydroxy-ethyl]phenyl}carbamates **7a**–**h** by nucleophilic addition of hydride anions (Scheme 2) using simple and convenient reduction with sodium borohydride [32]. The molecules **7a**–**h** were synthesized in 80% to 94% yields.

Detailed spectral characteristics (^1^H-NMR, ^13^C-NMR, HR-MS or ESI-MS) of the thirty-two prepared intermediates **3a**–**h**, **4**–**h**, **6a**–**h** and **7a**–**h**, are given in the Materials and Methods section of a current paper.

Addition of a saturated solution of hydrogen chloride in diethyl ether into a particular solution of the compounds **7a**–**h** in chloroform led to the desired 1-(2-{3-/4-[(alkoxycarbonyl)amino]phen-yl}-2-hydroxyethyl)-4-(2-fluorophenyl)piperazin-1-ium chlorides **8a**–**h**. The crude products **8a**–**h** were purified by recrystallization from acetone providing 84% to 96% yields (Table 1, Scheme 2).

Purity of the final salts **8a**–**h** was verified by thin-layer chromatography (TLC) using petroleum ether/diethyl amine eluant (10:3, *v*/*v*) as a mobile phase. Spots were observed under iodine vapours/UV light at a wavelength (*λ*) of 254 nm. The position and length of an 3-/4-alkoxycarbonylamino fragment influenced values of a retardation factor (*R*_f_), as expected. The 3-positional isomers **8a**–**d** showed slightly higher *R*_f_ values (*R*_f_ = 0.40–0.73) compared to those of the 4-substituted ones **8e**–**h** (0.31–0.56). The *R*_f_ values have been provided in detail in the Materials and Methods section of a current paper.

The newly synthesized target substances **8a**–**h** were fully characterized by their IR, ^1^H-NMR, ^13^C-NMR and ESI-MS spectral values, which were in full accordance with proposed structures. Analyzing the IR spectra of **8a**–**h**, bands typical for stretching vibrations *υ* (C = O) were observed in the region from 1730 cm**^−^**^1^ to 1718 cm**^−^**^1^. Identity of aromatic rings was confirmed by presence of *υ* (C = C) at around 1600 cm**^−^**^1^. The recorded IR spectra also afforded vibrations in the range from 1552 cm**^−^**^1^ to 1543 cm**^−^**^1^ due to *δ*(N–H). The bands between 1234 cm**^−^**^1^ and 1221 cm**^−^**^1^ were related to asymmetric stretching of a C–O–C fragment. The in-plane deformation vibrations (*δ*_ip_) at around 1020 cm**^−^**^1^ and out-of-plane deformation vibrations (*δ*_oop_) at around 850 cm**^−^**^1^ of a =C–H group were also observed.

In the ^1^H-NMR, signals of particular protons were verified on basis of their chemical shift (*δ*), multiplicities and coupling constants in DMSO-*d*_6_. Regarding the 3-alkoxycarbonylamino substituent-containing molecules **8a**–**d**, a proton signal of a carbamoyloxy group was detected in the *δ* interval from 9.60 ppm to 9.57 ppm. A shift of this chain to a 4-position (compounds **8e**–**h**) led to slightly higher *δ* values recognized from 9.71 ppm to 9.67 ppm. The *δ* chemical shift between 154.90 ppm and 153.25 ppm (doublet) was assigned to the carbon atom of a C–F bond in the ^13^C-NMR spectra of prepared salts **8a**–**h**. The carbon of a carbamoyloxy group was identified in the *δ* range from 153.91 ppm to 152.91 ppm.

Elemental analyses of the synthesized derivatives **8a**–**h** indicated that addition of a saturated solution of hydrogen chloride in diethyl ether caused a protonation of only one nitrogen of a piperazin-1,4-diyl fragment. This was due to a positive mesomeric effect of the nitrogen atom towards an aromatic ring. This conclusion was also evidenced by mass spectral values of these compounds, for which particular [M + H]^+^ molecular peaks were observed. Current elemental analyses results (% C, H, N) were within ±0.40% of theoretical values for the proposed monohydrochlorides.

#### 2.1.2. Lipohydrophilic Properties

Lipophilicity has been the physicochemical parameter continually attracting prime interest in *QSAR* and *SAR* studies as a predominant descriptor of pharmacodynamic, pharmacokinetic and toxic aspects of the antimycobacterial drugs [33,34,35,36].

A partition coefficient *P* (or its logarithm) between water or a phosphate buffer and octan-1-ol has been used as a preferential experimental expression of lipophilic properties of a compound. However, the log *P* parameter is losing that role as the method of a choice due to some methodological drawbacks and limitations, which were extensively described and explained in [37]. Chromatographic methods have been therefore developed and used successfully to estimate the lipophilicity of organic compounds [37].

Lipophilicity indices *R*_M_ and *k* (log *k*) of the compounds **8a**–**h** were estimated by the reversed-phase thin-layer chromatography (RP-TLC) and reversed-phase high-performance liquid chromatography (RP-HPLC). The reason for more detailed chromatographic characterization of the salts **8a**–**h** was their better solubility in polar media (mobile phases) compared to free bases **7a**–**h**.

In addition, previous _in vitro_ antimycobacterial assays [18] employed structurally very similar compounds **MM** as hydrochlorides (Figure 2). This approach was very beneficial due to improvement in their solubility in tested media compared to free bases.

Based on previous experience, it would be more precise to evaluate the lipohydrophilic properties and antimycobacterial activities of **8a**–**h** and compare the observed values to those related to the **MM** series.

Calculations of the *R*_M_ and log *k* parameters were detailed in the Materials and Methods section of a current paper.

In the RP-TLC, silica gel plates impregnated by a hand with a variously concentrated silicone oil in heptane (a strong hydrophobic agent) were used as a non-polar stationary phase [37]. Optimal differences in *R*_M_ values within both homological groups **8a**–**d** and **8e**–**h** were observed if 1% silicone oil in heptane was chosen (Tables 1 and S1 in Supplementary Materials).

The calculated *R*_M_ values for the **8a**–**d** series varied from −0.55 to 0.01, the molecules **8e**–**h** showed higher *R*_M_s from −0.02 to 0.63 (Table 1). These *R*_M_ parameters were considered at least useful as a “quick and rough” estimation of lipophilicity.

Octadecyl-functionalized silica gel was used as a stationary phase in the RP-HPLC evaluation of **8a**–**h**. A gradient of two solvents at different volume ratios modulated retention properties of a stationary phase [38]. Liquid binary mixtures of methanol (MeOH) with water were employed as mobile phases in a present isocratic RP-HPLC method. The MeOH organic modifier was preferred because of making a reversed-phase chromatographic system closer to the octan-1-ol/water partitioning one in terms of sensitivity to *H*-bond donor properties of investigated compounds [39,40]. The modifier was applied in different volume concentrations that varied from 60% to 85% (*v*/*v*).

The isocratic separation was possible and in addition, reasonable retention of the analyzed compounds **8a**–**h** was observed in all mobile phases. The estimated *k* parameters were found in an acceptable interval from 0.5 to 20 [39] and were listed in Table S2 (Supplementary Materials).

Increase in a volume concentration of MeOH led to shortening of *t*_r_ and log *k* values for all molecules **8a**–**h** (Table 1).

The 3-alkoxycarbonylamino substituent-containing derivatives **8a**–**d** showed higher *t*_r_ and log *k* parameters than their 4-positional isomers **8e**–**h** (Table 1), albeit excluding compounds **8d** and **8h**. Elongation of an *R* substituent led to the increase in *t*_r_ and log *k* values within both groups **8a**–**d** and **8e**–**h** (Table 1). Lower log *k* parameters of a molecule **8e** compared to those of a derivative **8a** (Table S2 in Supplementary Materials) would be a result of “linearity“ of the 4-substituted molecule as well as interactions between the mobile phase and methoxy moiety of **8e**. Hydrogen atoms of this group were more acidic due to movement of electrons as a consequence of a different electronegativity of carbon and oxygen so the ability of the compound **8e** to form hydrogen bonds with a particular MeOH-containing mobile phase would be enhanced.

The highest log *k* values were observed for 1-(2-{3-[(butoxycarbonyl)amino]phenyl}-2-hydr-oxyethyl)-4-(2-fluorophenyl)piperazin-1-ium chloride (**8d**) and its positional isomer, 1-(2-{4-[(butoxycarbonyl)amino]phenyl}-2-hydroxyethyl)-4-(2-fluorophenyl)piperazin-1-ium chloride (**8h**; Table 1).

Extrapolation of the estimated log *k* parameters to elution with 100% water, i.e., the calculation of log *k*_w_ values, has become a widely accepted approach. The log *k*_w_ descriptor has been considered more efficient predictor of a biological activity than the log *k* itself because it reduced influence of an organic mobile phase modifier what was necessary to obtain measurable elution [41,42,43]. The log *k*_w_ values were extrapolated from intercepts of a linear relationship between the log *k* and volume fraction of a mobile phase modifier (*ϕ*_M_) using a Snyder-Soczewiński relationship [42,43,44]. The linear relationship was justified by a correlation coefficient (*R*) > 0.9900 and adjusted coefficient of determination (*Adj. R*^2^) > 0.9700 for all fitting models, excluding the one connected with the compound **8d** (Table 2). Anyway, values of the calculated statistical descriptors related to **8d** were also satisfactory (*R* = 0.9730, *Adj. R*^2^ = 0.9201; Table 2).

The extrapolated log *k*_w_ values of the analyzed compounds **8a**–**h** (Table 2) were in accordance with their elution order and hydrophobicity and ranged from 2.113 (**8e**) to 2.930 (**8h**). Higher log *k*_w_ values were observed for the derivatives **8a**–**c** compared to **8e**–**g**. Butoxycarbonylamino substituent-containing compounds **8d** and **8h** were found to be the most lipophilic, as proven by their log *k*_w_ of 2.796 (**8d**) and 2.930 (**8h**), respectively (Table 2).

The slope *S* of a regression line used to obtain log *k*_w_ encoded notable information regarding a specific hydrophobic surface area and could serve as indicative measure of uniformity of a retention mechanism. If uniformity was observed, a convenient model between the slope(s) and intercept(s) was anticipated [45]. The currently calculated *S* parameters varied from 2.7386 (**8e**) to 3.3441 (**8h**; Table 2). The slope *S* was related to a specific hydrophobic surface of a compound and could be used as alternative measure of its lipophilicity [46].

Statistically extremely significant relationship between the log *k*_w_ and *S* values was described by Equation (1). The model was characterized by the *Prob > F* parameter, which was in the range from 0 to <0.0010:

*S* = 0.6457 ( ± 0.0882) × log *k*_w_ + 1.4219 ( ± 0.2208) (1)

*RSS* = 0.0199, *R* = 0.9483, *Adj. R*^2^ = 0.8826, *RMSE* = 0.0575, *NoR* = 0.1409, *F* = 53.61, *Prob > F* = 0.0003, *n* = 8

Based on the calculated statistical descriptors provided above, the uniformity of a retention mechanism of the studied derivatives **8a**–**h** was proven and suitability of selected mobile phases was confirmed for lipophilicity evaluation.

#### 2.1.3. Electronic Properties

Electronic properties of the inspected compounds **8a**–**h** (Table 3) were characterized by logarithms of molar absorption coefficients (log *ε*) of their methanolic solutions (*c* = 3.0 × 10^−5^ M) investigated in the UV/Vis region of the spectrum.

The solutions showed three absorption maxima in a near ultraviolet (quarz) region of the electromagnetic spectrum between 200 and 400 nm [47], e.g., *λ*_1_ = 208–210 nm, *λ*_2(Ch-T)_ = 238–240 nm and *λ*_3_ = 274–276 nm, respectively (Table 3).

The log *ε*_2(Ch-T)_ parameters of the compounds **8a**–**d** observed at a charge-transfer absorption maximum *λ*_2(Ch-T)_ were found in a narrow interval from 4.30 (**8a**) to 4.37 (**8c**). The methanolic solutions of **8e**–**h** were characterized by higher log *ε*_2(Ch-T)_ values than the ones of **8a**–**d** and varied from 4.42 (**8h**) to 4.67 (**8e**; Table 3). In addition, elongation of the 4-side chain led to lower log *ε* values related to all observed absorption maxima (Table 3).

### 2.2. Biological Assays

#### 2.2.1. _In Vitro_ Antimycobacterial Activity and Structure–Activity Relationships

The compounds **8a**–**h** were initially tested _in vitro_ against *M. tuberculosis* CNCTC My 331/88 (identical with H_37_R_v_ and ATCC 2794), *M. avium* CNCTC My 330/80 (identical with ATCC 25291), *M. avium intracellulare* ATCC 13950 and *M. kansasii* CNCTC My 235/80 (identical with ATCC 12478), respectively, as well as against *M. kansasii* 6 509/96, *M. kansasii* CIT11/06, *M. avium* subsp. *paratuberculosis* CIT03 and *M. avium hominissuis* CIT10/08 clinical isolates by methods described earlier [17,48,49,50].

The *MIC* was defined as the lowest concentration of a particular compound, which (i) inhibited growth of *M. tuberculosis* CNCTC My 331/88, *M. avium* CNCTC My 330/80, *M. kansasii* CNCTC My 235/80 or *M. kansasii* 6 509/96 [48]; (ii) prevented a visual colour change from blue to pink when testing susceptibility of the *M. kansasii* CIT11/06, *M. avium* subsp. *paratuberculosis* CIT03, *M. avium hominissuis* CIT10/08 or *M. avium intracellulare* ATCC 13950 strain. The *MIC* for given mycobacteria was defined as 90% or greater reduction of their growth (*IC*_90_) compared to a control [17,49,50].

The efficiency of newly synthesized molecules **8a**–**h** was compared to the activity of reference drugs, i.e., isonicotinic acid hydrazide (isoniazid, INH), ethambutol (EMB), ofloxacin (OFLX), ciprofloxacin (CPX) or pyrazinamide (PZA) under same experimental conditions.

Next sections of the current research were focused specifically on the most susceptible strains, i.e., *M. tuberculosis* CNCTC My 331/88, *M. kansasii* CNCTC My 235/80 and *M. kansasii* 6 509/96, respectively. The _in vitro_ activities (*MIC* values) of the most promising *N*-arylpiperazines were highlighted by a bold font style in gray (Table 4).

The position of a side chain notably affected the activity of tested derivatives **8a**–**h** against *M. tuberculosis* CNCTC My 331/88 (Table 4). After 14- and 21-day cultivation (14-d/21-d), the 4-positional isomers were more active, with the *MIC* values ranging from 8 μM (**8h**) to 125 μM (**8e**), than the 3-positional ones, which possessed the *MIC*s from 32 μM (**8d**) to 250 μM (**8a**).

Among all the _in vitro_ screened molecules, INH standard was found to be the most active with the *MIC* = 0.5 μM (14-d/21-d).

**Figure 3:**
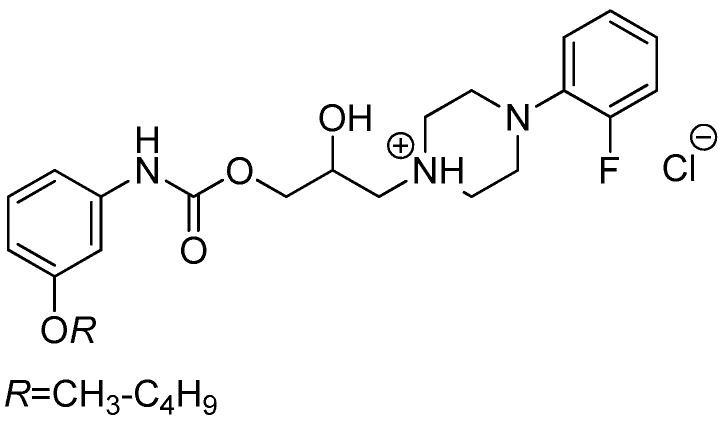
*N*-Arylpiperazines containing a 2-hydroxypropane-1,3-diyl connecting chain (a series **IM**), which were _in vitro_ screened against *M. tuberculosis* H_37_R_v_ [51].

Introduction of a 4-(pyrimidin-2-yl)piperazin-1-yl fragment instead of the 4-(2-fluorophen-yl)piperazin-1-yl one led to the derivatives **MM**, which showed a comparable efficiency [18] to the compounds **8h** and **8d**, especially if they contained *R* = C_3_H_7_/C_4_H_9_ (Figure 2). Similarly, presence of an 3-alkoxyphenylcarbamoyloxy moiety (alkoxy = methoxy to butoxy) and elongation of a connecting chain resulted in the molecules **IM** (Figure 3) with a comparable _in vitro_ activity [51] to **8a**–**h**.

Isosteric replacement of the carbamoyloxy with a carboxy group in a structure of the compounds **IM** and introduction of an 4-alkoxycarbonylamino side chain (alkoxy = methoxy to butoxy) at the aromatic ring resulted in decreased _in vitro_ efficiency of such modified racemic derivatives against *M. tuberculosis* H_37_R_v_ [16].

The compounds **8a**–**d** were more efficient against *M. kansasii* My 235/80 and *M. kansasii* 6 509/96 than the substances **8e**–**h**. The most active compound against both mycobacteria was **8d** with the *MIC* = 16 μM and 62.5 μM, respectively, depending on a particular strain and also on the number of days of incubation. Increase in length of the side chain resulted in lower *MIC* values of **8a**–**d** against both tested *M. kansasii* strains. The observed *MIC* values were, however, higher compared to the ones related to EMB with the *MIC* = 1 μM and 2 μM (14-d/21-d), or OFLX, which showed the *MIC* = 0.5 μM and 1 μM, respectively (14-d/21-d; Table 4).

The _in vitro_ activity of screened compounds **8a**–**h** against a non-tuberculous INH-resistant *M. avium* CNCTC My 330/80 was apparently dependent on the position of the alkoxycarbonylamino chain *R*. Its presence in the 3-position (**8a**–**d**) led to the *MIC* values varying from 62.5 μM (**8d**) to 500 μM (**8a**; 14-d/21-d). However, a potential of 4-substituent-containing derivatives (**8e**–**h**) to fight given *mycobacterium* was insufficient (*MIC* > 250 μM; Table 4).

The activity of the most active substance **8d** (*MIC* = 62.5 μM; 14-d/21-d) against *M. avium* CNCTC My 330/80 was comparable to the effectiveness of OFLX (*MIC* = 32 μM and 62.5 μM, respectively; 14-d/21-d). The EMB reference drug was slightly more active (*MIC* = 16 μM; 14-d/21-d) than **8d**. Elongation of an 3-*R* side chain led to more potent compounds (Table 4).

The molecules **8a**–**h** were _in vitro_ practically inactive against *M. kansasii* CIT11/06, *M. avium* subsp. *paratuberculosis* CIT03, *M. avium intracellulare* ATCC 13950 and *M. avium hominissuis* CIT10/08, respectively (*MIC* ≥ 295 μM; Table 5). The CPX standard was the most effective among all investigated compounds (*MIC* > 91 μM and 181 μM, respectively; Table 5).

The lipophilicity has been considered one of the most important factors, which critically influenced a compound´s activity in penetrating mycobacterial cell walls [48,52,53]. To explore this statement in detail, relationships between the log *k*_w_ (independent variable) and activity values (dependent variable) of the compounds **8a**–**h** were inspected. For purposes of the current *SAR* study, observed *MIC* values were transformed into log (1/*MIC* [M]) units.

The INH, EMB and OFLX standard drugs were not included in the investigated models because of being structurally different and, in addition, different modes of their action have been proposed [54,55,56,57,58,59,60].

Linear regression analyses were carried out using the Origin Pro 9.0.0 software (OriginLab Corporation, Northampton, MA, USA). More details about particular statistical parameters and significance levels were provided in the Materials and Methods section of a current paper.

Regarding the **8a**–**h** set, analyses related to *M. tuberculosis* My 331/88 (*M. tuberculosis* H_37_R_v_; 14-d/21-d) were expressed by Equations (2) and (3):

*MT* My 331/88 (14-d), **8a**–**h**:

log (1/*MIC* [M]) = 1.4383 ( ± 0.5893) × log *k*_w_ + 0.6071 ( ± 1.5239) (2)

*RSS* = 0.8867, *R* = 0.7059, *Adj. R*^2^ = 0.4146, *RMSE* = 0.3844, *NoR* = 0.9417, *F* = 5.95, *Prob > F* = 0.0504, *n* = 8

*MT* My 331/88 (21-d), **8a**–**h**:

log (1/*MIC* [M]) = 1.4819 ( ± 0.5598) × log *k*_w_ + 0.4586 ( ± 1.4476) (3)

*RSS* = 0.8002, *R* = 0.7340, *Adj. R*^2^ = 0.4619, *RMSE* = 0.3652, *NoR* = 0.8945, *F* = 7.01, *Prob > F* = 0.0382, *n* = 8

Based on given statistical parameters, only Equation (3) described a statistically significant model, for which the *Prob > F* value was found the interval from 0.0100 to <0.0500.

If the analysis was separately applied to the groups **8a**–**d** and **8e**–**h**, much more convenient values of statistical descriptors were calculated for **8a**–**d** (14-d/21-d) as follows: *RSS* = 0.0003, *R* = 0.9997, *Adj. R*^2^ = 0.9991, *RMSE* = 0.0119, *NoR* = 0.0168, *F* = 3143.04, *Prob > F* = 0.0003, *n* = 4. The statistically extremely significant models (Table S3 in Supplementary Materials) were characterized by Equations (S1) and (S3).

On the other hand, the relationships connected with the **8e**–**h** series were statistically insignificant, as proven by Equation (S2) and (S4), respectively (Table S3).

Focusing on the *M. kansasii* My 235/80 strain after 7-day cultivation (7-d), only the derivatives **8b**–**d** showed interesting *MIC* values of 32 μM or 62.5 μM (Table 4). However, the linear regression model involving the log *k*_w_ and log (1/*MIC* [M]) values related to the **8a**–**d** set was statistically insignificant, as expected (Equation (S5) in Table S3).

Differing effectacy of the compounds **8a**–**d** against *M. tuberculosis* My 331/88 (*M. tuberculosis* H_37_R_v_) and *M. kansasii* My 235/80 (Table 4) was probably a consequence of diverse composition of the bacterial membrane of those strains [61,62].

Lipophilicity could play a crucial role in a mechanism of action of the compounds **8a**–**d** against the *M. kansasii* 6 509/96 clinical isolate (7-d; Table 4), as provided by Equation (4).

*MK* 6 509/96 (7-d), **8a**–**d**:

log (1/*MIC* [M]) = 2.4098 (± 0.0576) × log *k*_w_ − 1.9466 ( ± 0.1507) (4)

*RSS* = 0.0005, *R* = 0.9994, *Adj. R*^2^ = 0.9982, *RMSE* = 0.0158, *NoR* = 0.0224, *F* = 1750.57, *Prob > F* = 0.0005, *n* = 4

Despite of very convenient values of the statistical descriptors, a main limitation of the approach was a low number of the compounds (*n* = 4) involved in this analysis.

The log *ε*_2(Ch-T)_ values (independent variable), which were observed at the charge-transfer absorption maximum *λ*_2(Ch-T)_, were taken into a special consideration (Table 3), because they could be the most sensitive to the differences in position and electronic properties of a particular alkoxycarbonylamino substituent [47].

**Figure 4:**
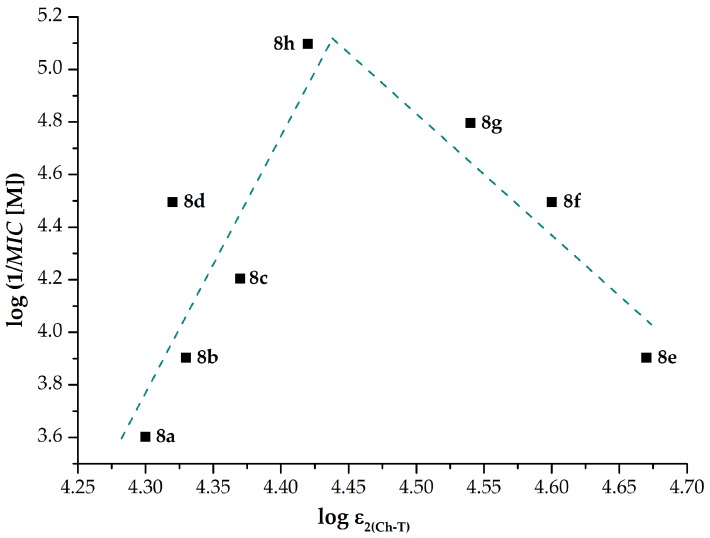
Bilinear relationship between the log *ε*_2(Ch-T)_ and log (1/*MIC* [M]) parameters of the investigated compounds **8a**–**h**. The dependent variable values were taken from the _in vitro_ screening of these derivatives against *M. tuberculosis* CNCTC My 331/88 (*M. tuberculosis* H_37_R_v_; 14-d).

The relationship between the log *ε*_2(Ch-T)_ parameters and log (1/*MIC* [M]) values connected with the _in vitro_ screening of **8a**–**h** against *M. tuberculosis* My 331/88 (*M. tuberculosis* H_37_R_v_; 14-d) provided a bilinear course. Based on this fitting, maximal efficiency of the tested compounds could be observed if their log *ε*_2(Ch-T) _values were approximately 4.43 (Figure 4).

Regarding the *M. kansasii* My 235/80 and *M. kansasii* 6 509/96 strains, no significant relationships between the _in vitro_ activity of the compounds **8a**–**h** and their electronic features were observed.

#### 2.2.2. _In Vitro_ Cytotoxicity Screening

Cytotoxicity of the compounds **8a**–**h** was inspected as the *LD*_50_ value, i.e., a lethal dose to 50% of a cell population, which was derived from survival rate curves. The highest dose of all tested derivatives in a medium (30 μM) did not lead to a significant lethal effect on a human monocytic leukemia THP-1 cell line.

The tested molecules showed low (insignificant) toxicity of *LD*_50_ > 30 μM against given cell line. Moreover, the compounds **8a** and **8e** increased proliferation of the THP-1 cells in 24 h when compared to a control. Relative survival rate (in percentages) of the THP-1 cells for all tested derivatives was found to be over 80%, excluding the molecule **8d**, where it was 79% at the highest tested concentration of 30 μM (Figure S1 in Supplementary Materials). Only the compounds with the *IC*_50_ < 10 μM could be considered antiproliferative (cytotoxic) agents [63], and the highest tested concentration used for the current toxicity assay was 3-fold this value.

As the *LD*_50_ values of oxaliplatin and camptothecin standard drugs assessed in this cell line were considerably lower (1.7 ± 0.64 μM and 0.16 ± 0.07 μM, respectively), the discussed compounds **8a**–**h** were deemed non-toxic agents suitable for further design and development of novel antimycobacterials.

## 3. Materials and Methods

### 3.1. General Information

All reagents used for syntheses were commercially available from Alpha Aesar (Lancashire, UK), Fluka Chemie (Buchs, Switzerland), Lachema (Brno, Czech Republic), LachNer (Neratovice, Czech Republic), Lancaster (Ward Hill, MA, USA), Merck (Darmstadt, Germany) or Sigma-Aldrich (Dorset, UK) in sufficient quality and were used without additional purification. Solvents were dried and freshly distilled before use.

Thin-layer chromatography (TLC) Kieselgel 60 F_254_ plates (Merck) visualized by UV irradiation (*λ* = 254 nm) were used to monitor reactions and purity of synthesized compounds.

Melting point (Mp or mp) values of prepared intermediates and final compounds, respectively, were determined on the Kofler hot plate apparatus HMK (Franz Kustner Nacht GK, Dresden, Germany) and left uncorrected. The mps of some intermediates were already published, i.e., **3a**: 102–104 °C [64]; **3b**: 111–112 °C [64] and 113–114 °C [65], respectively; **3d**: 53–55 °C [64]; **3e**: 168 °C [64] and 160–162 °C [66], respectively; **3f**: 158 °C [67], 157–158 °C [68,69] and 160–161 °C [64], respectively; **3h**: 87–88.5 °C [64]; **4a**: 99–103 °C [70]; **4b**: 108–110 °C [71]; **4d**: 80–86 °C [70]; **4e**: 200–201 °C [72].

The *R*_f_ values of prepared salts **8a**–**h** were obtained by the TLC on 10-cm aluminium sheets pre-coated with silica gel 60 F_254_ (0.25 mm thickness; Merck) in glass developing chambers using petroleum ether/diethylamine (10:3, *v*/*v*) eluant as a mobile phase. Spots were located under iodine vapours/UV light at *λ* = 254 nm.

The ^1^H- and ^13^C-NMR spectral analyses were carried out on the FT-NMR spectrometer (Varian Co., Palo Alto, CA, USA) operating at 300 MHz (^1^H-NMR) and 75 MHz (^13^C-NMR), respectively, in dried DMSO-*d*_6_ using tetramethylsilane (TMS; Sigma-Aldrich, Darmstadt, Germany) as an internal standard.

Chemical shifts were reported in a *δ* scale in parts *per* million (ppm), coupling constants *J* were given in Hertz (Hz) and spin multiplicities were expressed as follows: s (singlet), d (doublet), dd (double doublet), t (triplet), q (quartet), m (multiplet). A complete assignment of ^1^H- and ^13^C-NMR resonances was based on an interpretation of standard NMR values.

The FT-IR (IR) spectra were obtained by the ATR technique on the FT-IR spectrophotometer Impact 410 (Thermo Fisher Scientific, West Palm Beach, FL, USA). The absorption frequencies *ῦ*_max_ were reported in reciprocal centimeters (cm^−1^) in a recorded range from 4000 cm^−1^ to 400 cm^−1^.

The mass spectra (HR-MS) of prepared intermediates **3a**–**h**, **4a**–**h** and **6a**–**h** (Scheme 1 and Scheme 2) were measured using the high-performance liquid chromatograph Dionex UltiMate^®^ 3000 (Thermo Fischer Scientific) coupled with the LTQ Orbitrap XL^™^ Hybrid Ion Trap-Orbitrap Fourier Transform Mass Spectrometer (Thermo Fischer Scientific) with injection into HESI-II in a positive mode.

The liquid chromatography mass spectra of the compounds **7a**–**h** and **8a**–**h** (Scheme 2, Table 1) were carried out on the Agilent 1100 LC/MSD Trap (Agilent Technologies, Santa Clara, CA, USA) in a positive mode using electrospray ionization at atmospheric pressure. The particular compound was dissolved in methanol (*c* = 1 mg/mL) and the solution was passed through the XDB SOX 2.1 mm column (Agilent Technologies) with a 1.8 μm particle size at the pressure of 400 bar. The UV detection was performed at *λ* = 254 nm. Nebulization gas (N_2_) flow was 8 L/min, pressure was 40 psi. The MS electrospray operated at capillary voltage of 3.5 kV and temperature was set to 350 °C (ESI-MS). Fragments were described as a relationship between atomic mass units and charge (*m*/*z*), a recorded interval was from 50 *m*/*z* to 1000 *m*/*z*.

The elemental analysis (% C, H, N) of the compounds **8a**–**h** was carried out by the Perkin-Elmer 2400 Series-II Elemental Analyzer (Perkin-Elmer, Waltham, MA, USA) and all the derivatives were within ±0.40% of calculation.

The chromatographic HPLC-Diode Array Detection apparatus for the determination of capacity (retention) factor *k* (log *k*) values was the LC Agilent Infinity System (Agilent Technologies, Santa Clara, CA, USA) equipped with a Infinity 1260 gradient pump with a degasser, a 1260 HiPals automatic injector, a column thermostat 1290, a photo-diode array detector Infinity 1290 (all the equipments were obtained by Agilent Technologies) and personal computer with the Agilent ChemStation software (Agilent Technologies) for the registration of values and calibration procedures. The chromatographic column Eclipse plus RP C_18_, 150 × 4.6 mm i.d., a 5 μm particle size (Agilent Technologies), was used and thermostated at 35 °C.

The analyses were performed at pressure ranged from 7 MPa to 15 MPa. The detection wavelength was set to 260 nm, injection sample volume was 5 μL with a flow rate of 1.0 mL/min in all RP-HPLC analyses.

LC-MS Methanol (J. T. Baker Chemicals Co., Phillipsburg, NJ, USA) and HPLC-quality water (Sigma-Aldrich, Darmstadt, Germany) were used for a preparation of mobile phases. Water was firstly deionized and purified by the Millipore Simplicity 185 Ultrapure water purification system (Millipore, Billerica, MA, USA).

The UV/Vis spectra of methanolic solutions of the analyzed compounds **8a**–**h** (*c* = 3.0 × 10^−5^ M) were estimated on the 8452A Diode Array spectrophotometer HP-8452A (Hewlett Packard, Palo Alto, CA, USA). Methanol for UV-spectroscopy (Merck) was used for the preparation of these solutions.

Results of the UV/Vis analyses were collected and stored digitally using the ChemStation controller software (Agilent Technologies, Waldbronn, Germany). The HP-8452A apparatus measured a complete range of a spectrum from 190 nm to 820 nm.

### 3.2. Synthesis of Compounds

#### 3.2.1. General Procedure For the Preparation of Alkyl (3-/4-Acetylphenyl)carbamates (**3a**–**h**)

Into a stirred solution of 3-aminoacetophenone **1a** (CAS Registry Number 99-03-6; 5.00 g, 37 mmol) or 4-aminoacetophenone **1b** (CAS Registry Number 99-92-3; 5.00 g, 37 mmol) and pyridine (3.0 mL, 37 mmol) in 20 mL of acetone, a solution of methyl chloroformate **2a** (CAS Registry Number 79-22-1; 3.5 mL, 37 mmol), ethyl chloroformate **2b** (CAS Registry Number 541-41-3; 4.0 mL, 37 mmol), propyl chloroformate **2c** (CAS Registry Number 109-61-5; 4.5 mL, 37 mmol) or butyl chloroformate **2d** (CAS Registry Number 592-34-7; 5.0 mL, 37 mmol) in 5 mL of acetone, was added dropwise. The particular mixture was heated to reflux for 3 h [28]. When the reaction was completed (TLC control), the solvents were removed in vacuo, crude solid products **3a**–**h** were washed with distilled water and recrystallized from absolute ethanol. Full characterization data for the compounds **3a**–**h** (Scheme 1), isolated as colourless solids, are given below.

*Methyl (3-acetylphenyl)carbamate* (**3a**); CAS Registry Number 87743-55-3). Yield 6.80 g (95%); *M*_r_ 193.19; Mp 103–104 °C; ^1^H-NMR (DMSO-*d*_6_) *δ*_H_ (ppm): 9.87 (s, 1H, NHCOO), 8.06 (s, 1H, Ar–H), 7.71 (d, 1H, Ar–H, *J* = 7.7 Hz), 7.61 (d, 1H, Ar–H, *J* = 7.3 Hz), 7.44 (t, 1H, Ar–H, *J* = 8.2 Hz), 3.69 (s, 3H, COOCH_3_), 2.55 (s, 3H, COCH_3_); ^13^C-NMR (DMSO-*d*_6_) *δ*_C_ (ppm): 197.47, 153.92, 139.49, 137.37, 129.01, 122.56, 122.36, 117.26, 51.53, 26.51. HR-MS: for C_10_H_11_O_3_N [M – H]^+^ calculated 192.06552 *m*/*z*, found 192.06728 *m*/*z*.

*Ethyl (3-acetylphenyl)carbamate* (**3b**; CAS Registry Number 39569-24-9). Yield 7.50 g (98%); *M*_r_ 207.19; Mp 112–113 °C; ^1^H-NMR (DMSO-*d*_6_) *δ*_H_ (ppm): 9.83 (s, 1H, NHCOO), 8.07 (s, 1H, Ar–H), 7.68 (d, 1H, Ar–H, *J* = 8.1 Hz), 7.60 (d, 1H, Ar–H, *J* = 7.7 Hz), 7.42 (t, 1H, Ar–H, *J* = 7.8 Hz), 4.14 (q, 2H, CH_2_CH_3_, *J* = 7.1 Hz), 2.53 (s, 3H, COCH_3_), 1.24 (t, 3H, CH_2_CH_3_, *J* = 7.1 Hz ); ^13^C-NMR (DMSO-*d*_6_) *δ*_C_ (ppm): 197.53, 153.50, 139.60, 137.35, 129.01, 122.60, 122.33, 117.25, 60.21, 26.57, 14.37. HR-MS: for C_11_H_13_O_3_N [M – H]^+^ calculated 206.08117 *m*/*z*, found 206.08297 *m*/*z*.

*Propyl (3-acetylphenyl)carbamate* (**3c**). Yield 7.30 g (89%); *M*_r_ 221.19; Mp 101–103 °C; ^1^H-NMR (DMSO-*d*_6_) *δ*_H_ (ppm): 9.85 (s, 1H, NHCOO), 8.09 (s, 1H, Ar–H), 7.70 (d, 1H, Ar–H, *J* = 8.1 Hz), 7.61 (d, 1H, Ar–H, *J* = 8.1 Hz), 7.44 (t, 1H, Ar–H, *J* = 7.9 Hz), 4.06 (t, 2H, CH_2_CH_2_CH_3_, *J* = 6.6 Hz), 2.53 (s, 3H, COCH_3_), 1.72–1.56 (m, 2H, CH_2_CH_2_CH_3_), 0.94 (t, 3H, CH_2_CH_2_CH_3_, *J* = 7.5 Hz); ^13^C-NMR (DMSO-*d*_6_) *δ*_C_ (ppm): 197.45, 153.59, 139.60, 137.36, 128.96, 122.57, 122.27, 117.28, 65.73, 26.51, 21.76, 10.06. HR-MS: for C_12_H_15_O_3_N [M – H]^+^ calculated 220.09682 *m*/*z*, found 220.09854 *m*/*z*.

*Butyl (3-acetylphenyl)carbamate* (**3d**; CAS Registry Number 72531-03-4). Yield 7.90 g (91%); *M*_r_ 235.19; Mp 58–59 °C; ^1^H-NMR (DMSO-*d*_6_) *δ*_H_ (ppm): 9.83 (s, 1H, NHCOO), 8.07 (s, 1H, Ar–H), 7.68 (d, 1H, Ar–H, *J* = 7.7 Hz), 7.60 (d, 1H, Ar–H, *J* = 7.7 Hz), 7.42 (t, 1H, Ar–H, *J* = 7.9 Hz), 4.09 (t, 2H, CH_2_CH_2_CH_2_CH_3_, *J* = 6.6 Hz), 2.54 (s, 3H, COCH_3_), 1.64–1.54 (m, 2H, CH_2_CH_2_CH_2_CH_3_), 1.46–1.33 (m, 2H, CH_2_CH_2_CH_2_CH_3_), 0.92 (t, 3H, CH_2_CH_2_CH_2_CH_3_, *J* = 6.2 Hz); ^13^C-NMR (DMSO-*d*_6_) *δ*_C_ (ppm): 197.53, 153.60, 139.60, 137.35, 129.01, 122.59, 122.30, 117.28, 63.94, 30.46, 26.56, 18.49, 13.45. HR-MS: for C_13_H_17_O_3_N [M – H]^+^ calculated 234.11247 *m*/*z*, found 234.11425 *m*/*z*.

*Methyl (4-acetylphenyl)carbamate* (**3e**; CAS Registry Number 60677-43-2). Yield 7.05 g (99%); *M*_r_ 193.19; Mp 168–170 °C; ^1^H-NMR (DMSO-*d*_6_) *δ*_H_ (ppm): 10.09 (s, 1H, NHCOO), 7.91 (d, 2H, Ar–H, *J* = 8.8 Hz), 7.59 (d, 2H, Ar–H, *J* = 8.8 Hz), 3.70 (s, 3H, COOCH_3_), 2.52 (s, 3H, COCH_3_); ^13^C-NMR (DMSO-*d*_6_) *δ*_C_ (ppm): 196.28, 153.72, 143.60, 130.99, 129.43, 117.20, 51.79, 26.24. HR-MS: for C_10_H_11_O_3_N [M – H]^+^ calculated 192.06552 *m*/*z*, found 192.06728 *m*/*z*.

*Ethyl (4-acetylphenyl)carbamate* (**3f**; CAS Registry Number 5520-79-6). Yield 7.60 g (99%); *M*_r_ 207.19; Mp 161–163 °C; ^1^H-NMR (DMSO-*d*_6_) *δ*_H_ (ppm): 10.08 (s, 1H, NHCOO), 7.91 (d, 2H, Ar–H, *J* = 8.8 Hz), 7.59 (d, 2H, Ar–H, *J* = 8.8 Hz), 4.13 (q, 2H, CH_2_CH_3_, *J* = 7.1 Hz), 2.52 (s, 3H, COCH_3_), 1.24 (t, 3H, CH_2_CH_3_, *J* = 7.1 Hz); ^13^C-NMR (DMSO-*d*_6_) *δ*_C_ (ppm): 196.28, 153.38, 143.73, 130.94, 129.42, 117.19, 60.44, 26.23, 14.39. HR-MS: for C_11_H_13_O_3_N [M – H]^+^ calculated 206.08117 *m*/*z*, found 206.08295 *m*/*z*.

*Propyl (4-acetylphenyl)carbamate* (**3g**). Yield 8.05 g (98%); *M*_r_ 221.19; Mp 125–126 °C; ^1^H-NMR (DMSO-*d*_6_) *δ*_H_ (ppm): 10.07 (s, 1H, NHCOO), 7.91 (d, 2H, Ar–H, *J* = 8.8 Hz), 7.60 (d, 2H, Ar–H, *J* = 8.8 Hz), 4.07 (t, 2H, CH_2_CH_2_CH_3_, *J* = 6.6 Hz), 2.51 (s, 3H, COCH_3_), 1.75–1.57 (m, 2H, CH_2_CH_2_CH_3_), 0.94 (t, 3H, CH_2_CH_2_CH_3_, *J* = 7.3 Hz); ^13^C-NMR (DMSO-*d*_6_) *δ*_C_ (ppm): 196.28, 153.37, 143.71, 130.93, 129.42, 117.20, 65.96, 26.22, 21.73, 10.11. HR-MS: for C_12_H_15_O_3_N [M – H]^+^ calculated 220.09682 *m*/*z*, found 220.09852 *m*/*z*.

*Butyl (4-acetylphenyl)carbamate* (**3h**; CAS Registry Number 72531-04-5). Yield 8.23 g (95%); *M*_r_ 235.19; Mp 89–91 °C; ^1^H-NMR (DMSO-*d*_6_) *δ*_H_ (ppm): 10.05 (s, 1H, NHCOO), 7.91 (d, 2H, Ar–H, *J* = 8.8 Hz), 7.60 (d, 2H, Ar–H, *J* = 8.8 Hz), 4.11 (t, 2H, CH_2_CH_2_CH_2_CH_3_, *J* = 6.6 Hz), 2.51 (s, 3H, COCH_3_), 1.69–1.55 (m, 2H, CH_2_CH_2_CH_2_CH_3_), 1.48–1.30 (m, 2H, CH_2_CH_2_CH_2_CH_3_), 0.92 (t, 3H, CH_2_CH_2_CH_2_CH_3_, *J* = 7.3 Hz); ^13^C-NMR (DMSO-*d*_6_) *δ*_C_ (ppm): 196.27, 153.36, 143.69, 130.92, 129.40, 117.17, 64.14, 30.40, 26.22, 18.47, 13.45. HR-MS: for C_13_H_17_O_3_N [M – H]^+^ calculated 234.11247 *m*/*z*, found 234.11425 *m*/*z*.

#### 3.2.2. General Procedure For the Preparation of Alkyl [3-/4-(Bromoacetyl)phenyl]carbamates (**4a**–**h**)

Into a stirred solution of a particular alkyl (3-/4-acetylphenyl)carbamate, i.e., **3a**, **3e** (6.96 g, 36 mmol), **3b**, **3f** (7.46 g, 36 mmol), **3c**, **3g** (7.97 g, 36 mmol), **3d** or **3h** (8.47 g, 36 mmol), in 80 mL of chloroform, a solution of bromine (1.9 mL, 36 mmol) in 10 mL of chloroform was added dropwise and stirred for 3 h at laboratory temperature. When the reaction was completed (TLC control), the solvent was removed *in vacuo* giving a crude solid product [31]. Intermediates **4a**–**h** (Scheme 1) were recrystallized from propan-2-ol. Full characterization parameters for the compounds **4a**–**h**, isolated as colourless solids, are given below.

*Methyl [3-(bromoacetyl)phenyl]carbamate* (**4a**). Yield 7.40 g (75%); *M*_r_ 272.09; Mp 99–103 °C; ^1^H-NMR (DMSO-*d*_6_) *δ*_H_ (ppm): 9.92 (s, 1H, NHCO), 8.08 (s, 1H, Ar–H), 7.78–7.66 (m, 2H, Ar–H), 7.47 (t, 1H, Ar–H, *J* = 8.1 Hz), 4.89 (s, 2H, COCH_2_Br), 3.68 (s, 3H, COOCH_3_); ^13^C-NMR (DMSO-*d*_6_) *δ*_C_ (ppm): 191.39, 153.92, 139.70, 134.51, 129.17, 123.28, 122.98, 117.62, 51.64, 33.61. HR-MS: for C_10_H_10_O_3_BrN [M – H]^+^ calculated 269.97603 *m*/*z*, found 269.97781 *m*/*z*.

*Ethyl [3-(bromoacetyl)phenyl]carbamate* (**4b**; CAS Registry Number 88541-97-3).Yield 9.30 g (90%); *M*_r_ 286.09; Mp 105–108 °C; ^1^H-NMR (DMSO-*d*_6_) *δ*_H_ (ppm): 9.89 (s, 1H, NHCO), 8.10 (s, 1H, Ar–H), 7.78–7.66 (m, 2H, Ar–H), 7.46 (t, 1H, Ar–H, *J* = 7.7 Hz), 4.88 (s, 2H, COCH_2_Br), 4.14 (q, 2H, CH_2_CH_3_, *J* = 7.3 Hz), 1.25 (t, 3H, CH_2_CH_3_, *J* = 7.3 Hz); ^13^C-NMR (DMSO-*d*_6_) *δ*_C_ (ppm): 191.43, 153.48, 139.67, 134.51, 129.17, 123.29, 122.95, 117.60, 60.27, 33.72, 14.36. HR-MS: for C_11_H_12_O_3_BrN [M – H]^+^ calculated 283.99168 *m*/*z*, found 283.99340 *m*/*z*.

*Propyl [3-(bromoacetyl)phenyl]carbamate* (**4c**). Yield 9.60 g (89%); *M*_r_ 300.09; Mp 104–107 °C; ^1^H-NMR (DMSO-*d*_6_) *δ*_H_ (ppm): 9.90 (s, 1H, NHCO), 8.10 (s, 1H, Ar–H), 7.78–7.65 (m, 2H, Ar–H), 7.46 (t, 1H, Ar–H, *J* = 7.9 Hz), 4.88 (s, 2H, COCH_2_Br), 4.05 (t, 2H, CH_2_CH_2_CH_3_, *J* = 6.6 Hz), 1.73–1.55 (m, 2H, CH_2_CH_2_CH_3_), 0.93 (t, 3H, CH_2_CH_2_CH_3_, *J* = 7.3 Hz); ^13^C-NMR (DMSO-*d*_6_) *δ*_C_ (ppm): 191.43, 153.59, 139.81, 134.51, 129.17, 123.30, 122.93, 117.61, 65.81, 33.70, 21.76, 10.13. HR-MS: for C_12_H_14_O_3_BrN [M – H]^+^ calculated 298.00733 *m*/*z*, found 298.00913 *m*/*z*.

*Butyl [3-(bromoacetyl)phenyl]carbamate* (**4d**). Yield 9.60 g (85%); *M*_r_ 314.19; Mp 82–85 °C; ^1^H-NMR (DMSO-*d*_6_) *δ*_H_ (ppm): 9.89 (s, 1H, NHCO), 8.10 (s, 1H, Ar–H), 7.77–7.65 (m, 2H, Ar–H), 7.46 (t, 1H, Ar–H, *J* = 7.9 Hz), 4.88 (s, 2H, COCH_2_Br), 4.10 (t, 2H, CH_2_CH_2_CH_2_CH_3_, *J* = 6.6 Hz), 1.64–1.54 (m, 2H, CH_2_CH_2_CH_2_CH_3_), 1.47–1.34 (m, 2H, CH_2_CH_2_CH_2_CH_3_), 0.92 (t, 3H, CH_2_CH_2_CH_2_CH_3_, *J* = 7.3 Hz); ^13^C-NMR (DMSO-*d*_6_) *δ*_C_ (ppm): 191.41, 153.57, 139.79, 134.50, 129.16, 123.27, 122.91, 117.61, 63.99, 33.64, 30.43, 18.46, 13.43. HR-MS: for C_13_H_16_O_3_BrN [M – H]^+^ calculated 312.02298 *m*/*z*, found 312.02474 *m*/*z*.

*Methyl [4-(bromoacetyl)phenyl]carbamate* (4e; CAS Registry Number 942316-98-5). Yield 7.71 g (79%); *M*_r_ 272.09; Mp 200–202 °C; ^1^H-NMR (DMSO-*d*_6_) *δ*_H_ (ppm): 10.17 (s, 1H, NHCOO), 7.97 (d, 2H, Ar–H, *J* = 8.8 Hz), 7.61 (d, 2H, Ar–H, *J* = 8.8 Hz), 4.85 (s, 2H, COCH_2_Br), 3.71 (s, 3H, COOCH_3_); ^13^C-NMR (DMSO-*d*_6_) *δ*_C_ (ppm): 190.15, 153.71, 144.30, 130.17, 127.90, 117.32, 51.90, 33.46. HR-MS: for C_10_H_10_O_3_BrN [M – H]^+^ calculated 269.97603 *m*/*z*, found 269.97781 *m*/*z*.

*Ethyl [4-(bromoacetyl)phenyl]carbamate* (**4f**). Yield 9.63 g (93%); *M*_r_ 286.09; Mp 174–176 °C; ^1^H-NMR (DMSO-*d*_6_) *δ*_H_ (ppm): 10.14 (s, 1H, NHCOO), 7.96 (d, 2H, Ar–H, *J* = 8.8 Hz), 7.62 (d, 2H, Ar–H, *J* = 8.8 Hz), 4.85 (s, 2H, COCH_2_Br), 4.17 (q, 2H, CH_2_CH_3_, *J* = 6.7 Hz), 1.27 (t, 3H, CH_2_CH_3_, *J* = 7.3 Hz); ^13^C-NMR (DMSO-*d*_6_) *δ*_C_ (ppm): 190.12, 153.22, 144.38, 130.14, 127.81, 117.29, 60.56, 33.46, 14.31. HR-MS: for C_11_H_12_O_3_BrN [M – H]^+^ calculated 283.99168 *m*/*z*, found 283.9938 *m*/*z*.

*Propyl [4-(bromoacetyl)phenyl]carbamate* (**4g**). Yield 9.26 g (86%); *M*_r_ 300.09; Mp 154–155 °C; ^1^H-NMR (DMSO-*d*_6_) *δ*_H_ (ppm): 10.14 (s, 1H, NHCOO), 7.96 (d, 2H, Ar–H, *J* = 8.8 Hz), 7.62 (d, 2H, Ar–H, *J* = 8.8 Hz), 4.84 (s, 2H, COCH_2_Br), 4.08 (t, 2H, CH_2_CH_2_CH_3_, *J* = 6.6 Hz), 1.75–1.57 (m, 2H, CH_2_CH_2_CH_3_), 0.94 (t, 3H, CH_2_CH_2_CH_3_, *J* = 7.3 Hz); ^13^C-NMR (DMSO-*d*_6_) *δ*_C_ (ppm): 190.15, 153.36, 144.41, 130.16, 127.82, 117.32, 66.07, 33.48, 21.73, 10.14. HR-MS: for C_12_H_14_O_3_BrN [M – H]^+^ calculated 298.00733 *m*/*z*, found 298.00911 *m*/*z*.

*Butyl [4-(bromoacetyl)phenyl]carbamate* (**4h**). Yield 9.64 g (85%); *M*_r_ 314.19; Mp 152–154 °C; ^1^H-NMR (DMSO-*d*_6_) *δ*_H_ (ppm): 10.13 (s, 1H, NHCOO), 7.96 (d, 2H, Ar–H, *J* = 8.8 Hz), 7.62 (d, 2H, Ar–H, *J* = 8.8 Hz), 4.84 (s, 2H, COCH_2_Br), 4.12 (t, 2H, CH_2_CH_2_CH_2_CH_3_, *J* = 6.6 Hz), 1.70–1.56 (m, 2H, CH_2_CH_2_CH_2_CH_3_), 1.48–1.30 (m, 2H, CH_2_CH_2_CH_2_CH_3_), 0.92 (t, 3H, CH_2_CH_2_CH_2_CH_3_, *J* = 7.3 Hz); ^13^C-NMR (DMSO-*d*_6_) *δ*_C_ (ppm): 190.12, 153.31, 144.38, 130.13, 127.79, 117.28, 64.23, 33.43, 30.38, 18.47, 13.45. HR-MS: for C_13_H_16_O_3_BrN [M – H]^+^ 312.02298 *m*/*z*, found 312.02474 *m*/*z*.

#### 3.2.3. General Procedure For the Preparation of Alkyl {3-/4-[(4-(2-Fluorophenyl)piperazin-1-yl)-acetyl]phenyl}carbamates (**6a**–**h**)

A solution of a particular alkyl [3-/4-(bromoacetyl)phenyl]carbamate, i.e., **4a**, **4e** (1.50 g, 5.5 mmol), **4b**, **4f** (1.57 g, 5.5 mmol), **4c**, **4g** (1.65 g, 5.5 mmol), **4d** or **4h** (1.73 g, 5.5 mmol) in 30 mL of anhydrous tetrahydrofuran (THF) was added dropwise into a stirred solution of 1-(2-fluorophenyl)piperazine **5** (CAS Registry Number 111-15-0; 1.00 g, 5.5 mmol) and triethylamine (TEA; 0.8 mL, 5.5 mmol) in 20 mL of anhydrous THF [32]. The particular mixture was stirred for 3 h at laboratory temperature. When the reaction was completed (TLC control), the solvents were removed *in vacuo* and remaining solid was treated with 100 mL of distilled water and 100 mL of chloroform. The organic layer was washed with distilled water, dried over anhydrous sodium sulphate and evaporated *in vacuo* giving crude solid products. Prepared intermediates **6a**–**h** (Scheme 2) were recrystallized from acetone. Full characterization parameters for the compounds **6a**–**h**, isolated as colourless solids, are provided below.

*Methyl {3-[(4-(2-fluorophenyl)piperazin-1-yl)acetyl]phenyl}carbamate* (**6a**). Yield 1.80 g (86%); *M*_r_ 371.39; Mp 103–105 °C; ^1^H-NMR (DMSO-*d*_6_) *δ*_H_ (ppm): 9.86 (s, 1H, NHCO), 8.09 (s, 1H, Ar–H), 7.75–7.60 (m, 2H, Ar–H), 7.40 (t, 1H, Ar–H, *J* = 7.9 Hz), 7.07–6.98 (m, 4H, Ar–H), 3.87 (s, 2H, COCH_2_N), 3.68 (s, 3H, COOCH_3_), 3.10–3.00 (m, 4H, 3,5-piperazine), 2.75–2.60 (m, 4H, 2,6-piperazine); ^13^C-NMR (DMSO-*d*_6_) *δ*_C_ (ppm): 196.47, 154.86 (d, *J* = 242.8 Hz), 153.92, 139.76 (d, *J* = 8.4 Hz), 139.45, 136.44, 128.89, 124.66 (d, *J* = 3.0 Hz), 122.63, 122.19, 122.09 (d, *J* = 7.6 Hz), 119.15 (d, *J* = 2.3 Hz), 117.35, 115.37 (d, *J* = 20.5 Hz), 63.55, 52.56, 51.58, 50.00 (d, *J* = 3.1 Hz). HR-MS: for C_20_H_22_O_3_FN_3_ [M – H]^+^ calculated 370.15615 *m*/*z*, found 370.15791 *m*/*z*.

*Ethyl {3-[(4-(2-fluorophenyl)piperazin-1-yl)acetyl]phenyl}carbamate* (**6b**). Yield 1.64 g (75%); *M*_r_ 385.44; Mp 127–129 °C; ^1^H-NMR (DMSO-*d*_6_) *δ*_H_ (ppm): 9.84 (s, 1H, NHCO), 8.11 (s, 1H, Ar–H), 7.75–7.60 (m, 2H, Ar–H), 7.42 (t, 1H, Ar–H, *J* = 7.9 Hz), 7.10–6.94 (m, 4H, Ar–H), 4.13 (q, 2H, CH_2_CH_3_, *J* = 7.1 Hz), 3.87 (s, 2H, COCH_2_N), 3.10–3.00 (m, 4H, 3,5-piperazine), 2.75–2.60 (m, 4H, 2,6-piperazine), 1.24 (t, 3H, CH_2_CH_3_, *J* = 7.0 Hz); ^13^C-NMR (DMSO-*d*_6_) *δ*_C_ (ppm): 196.47, 154.89 (d, *J* = 242.8 Hz), 153.50, 139.77 (d, *J* = 8.4 Hz), 139.55, 136.41, 128.92, 124.68 (d, *J* = 3.0 Hz), 122.66, 122.15, 122.15 (d, *J* = 7.6 Hz), 119.17 (d, *J* = 2.3 Hz), 117.32, 115.78 (d, *J* = 20.5 Hz), 63.55, 60.21, 52.58, 49.99 (d, *J* = 3.1 Hz), 14.37. HR-MS: for C_21_H_24_O_3_FN_3_ [M – H]^+^ calculated 384.17180 *m*/*z*, found 384.17360 *m*/*z.*.

*Propyl {3-[(4-(2-fluorophenyl)piperazin-1-yl)acetyl]phenyl}carbamate* (**6c**). Yield 1.78 g (80%); *M*_r_ 399.47; Mp 124–126 °C; ^1^H-NMR (DMSO-*d*_6_) *δ*_H_ (ppm): 9.84 (s, 1H, NHCO), 8.11 (s, 1H, Ar–H), 7.75–7.60 (m, 2H, Ar–H), 7.42 (t, 1H, Ar–H, *J* = 7.9 Hz), 7.10–6.94 (m, 4H, Ar–H), 4.06 (t, 2H, CH_2_CH_2_CH_3_, *J* = 6.6 Hz), 3.87 (s, 2H, COCH_2_N), 3.10–3.00 (m, 4H, 3,5-piperazine), 2.70–2.55 (m, 4H, 2,6-piperazine), 1.74–1.56 (m, 2H, CH_2_CH_2_CH_3_), 0.94 (t, 3H, CH_2_CH_2_CH_3_, *J* = 7.3 Hz); ^13^C-NMR (DMSO-*d*_6_) *δ*_C_ (ppm): 196.47, 154.89 (d, *J* = 242.8 Hz), 153.62, 139.79 (d, *J* = 8.4 Hz), 139.58, 136.41, 128.95, 124.74 (d, *J* = 3.0 Hz), 122.66, 122.15 (d, *J* = 7.6 Hz), 122.16, 119.17 (d, *J* = 2.3 Hz), 117.32, 115.80 (d, *J* = 20.5 Hz), 65.77, 63.56, 52.61, 49.98 (d, *J* = 3.1 Hz), 21.79, 10.14. HR-MS: for C_22_H_26_O_3_FN_3_ [M – H]^+^ calculated 398.18745 *m*/*z*, found 398.18917 *m*/*z*.

*Butyl {3-[(4-(2-fluorophenyl)piperazin-1-yl)acetyl]phenyl}carbamate* (**6d**). Yield 1.85 g (81%); *M*_r_ 413.50; Mp 112–114 °C; ^1^H-NMR (DMSO-*d*_6_) *δ*_H_ (ppm): 9.84 (s, 1H, NHCO), 8.11 (s, 1H, Ar–H), 7.75–7.60 (m, 2H, Ar–H), 7.42 (t, 1H, Ar–H, *J* = 7.9 Hz), 7.11–6.94 (m, 4H, Ar–H), 4.10 (t, 2H, CH_2_CH_2_CH_2_CH_3_, *J* = 6.2 Hz), 3.87 (s, 2H, COCH_2_N), 3.15–3.00 (m, 4H, 3,5-piperazine), 2.80–2.60 (m, 4H, 2,6-piperazine), 1.68–1.55 (m, 2H, CH_2_CH_2_CH_2_CH_3_), 1.48–1.33 (m, 2H, CH_2_CH_2_CH_2_CH_3_), 0.91 (t, 3H, CH_2_CH_2_CH_2_CH_3_, *J* = 6.8 Hz); ^13^C-NMR (DMSO-*d*_6_) *δ*_C_ (ppm): 196.47, 154.89 (d, *J* = 242.8 Hz), 153.59, 139.77 (d, *J* = 8.4 Hz), 139.55, 136.43, 128.87, 124.65 (d, *J* = 3.0 Hz), 122.63, 122.10, 122.10 (d, *J* = 7.6 Hz), 119.13 (d, *J* = 2.3 Hz), 117.35, 115.75 (d, *J* = 20.5 Hz), 63.93, 63.56, 52.58, 49.98 (d, *J* = 3.1 Hz), 30.46, 18.47, 13.42. HR-MS: for C_23_H_28_O_3_FN_3_ [M – H]^+^ calculated 412.20310 *m*/*z*, found 412.20488 *m*/*z*.

*Methyl {4-[(4-(2-fluorophenyl)piperazin-1-yl)acetyl]phenyl}carbamate* (**6e**). Yield 1.71 g (81%); *M*_r_ 371.39; Mp 178–180 °C; ^1^H-NMR (DMSO-*d*_6_) *δ*_H_ (ppm): 10.09 (s, 1H, NHCO), 7.97 (d, 2H, Ar–H, *J* = 8.4 Hz), 7.59 (d, 2H, Ar–H, *J* = 8.4 Hz), 7.17–6.91 (m, 4H, Ar–H), 3.84 (s, 2H, COCH_2_N), 3.70 (s, 3H, COOCH_3_), 3.05–2.95 (m, 4H, 3,5-piperazine), 2.75–2.60 (m, 4H, 2,6-piperazine); ^13^C-NMR (DMSO-*d*_6_) *δ*_C_ (ppm): 195.13, 154.85 (d, *J* = 242.8 Hz), 153.66, 143.63, 139.70 (d, *J* = 8.4 Hz), 129.92, 129.39, 124.62 (d, *J* = 3.0 Hz), 122.10 (d, *J* = 7.6 Hz), 119.14 (d, *J* = 2.3 Hz), 117.14, 115.74 (d, *J* = 20.5 Hz), 63.32, 52.56, 51.73, 49.89 (d, *J* = 3.1 Hz). HR-MS: for C_20_H_22_O_3_FN_3_ [M – H]^+^ calculated 370.15615 *m*/*z*, found 370.15787 *m*/*z*.

*Ethyl {4-[(4-(2-fluorophenyl)piperazin-1-yl)acetyl]phenyl}carbamate* (**6f**). Yield 1.82 g (86%); *M*_r_ 385.44; Mp 172–174 °C; ^1^H-NMR (DMSO-*d*_6_) *δ*_H_ (ppm): 10.05 (s, 1H, NHCO), 7.96 (d, 2H, Ar–H, *J* = 8.4 Hz), 7.59 (d, 2H, Ar–H, *J* = 8.4 Hz), 7.17–6.91 (m, 4H, Ar–H), 4.13 (q, 2H, CH_2_CH_3_, *J* = 7.0 Hz), 3.82 (s, 2H, COCH_2_N), 3.05–2.95 (m, 4H, 3,5-piperazine), 2.75–2.60 (m, 4H, 2,6-piperazine), 1.25 (t, 3H, CH_2_CH_3_, *J* = 7.0 Hz); ^13^C-NMR (DMSO-*d*_6_) *δ*_C_ (ppm): 195.27, 154.85 (d, *J* = 242.8 Hz), 153.21, 143.69, 139.73 (d, *J* = 8.4 Hz), 129.92, 129.36, 124.62 (d, *J* = 3.0 Hz), 122.13 (d, *J* = 7.6 Hz), 119.14 (d, *J* = 2.3 Hz), 117.13, 115.76 (d, *J* = 20.5 Hz), 63.38, 60.39, 52.58, 49.94 (d, *J* = 3.1 Hz), 14.25. HR-MS: for C_21_H_24_O_3_FN_3_ [M – H]^+^ calculated 384.17180 *m*/*z*, found 384.17360 *m*/*z*.

*Propyl {4-[(4-(2-fluorophenyl)piperazin-1-yl)acetyl]phenyl}carbamate* (**6g**). Yield 1.71 g (75%); *M*_r_ 399.47; Mp 140–142 °C; ^1^H-NMR (DMSO-*d*_6_) *δ*_H_ (ppm): 10.06 (s, 1H, NHCO), 7.96 (d, 2H, Ar–H, *J* = 8.4 Hz), 7.59 (d, 2H, Ar–H, *J* = 8.4 Hz), 7.17–6.91 (m, 4H, Ar–H), 4.06 (t, 2H, CH_2_CH_2_CH_3_, *J* = 6.6 Hz), 3.83 (s, 2H, COCH_2_N), 3.05–2.95 (m, 4H, 3,5-piperazine), 2.75–2.60 (m, 4H, 2,6-piperazine), 1.75–1.57 (m, 2H, CH_2_CH_2_CH_3_), 0.93 (t, 3H, CH_2_CH_2_CH_3_, *J* = 7.3 Hz); ^13^C-NMR (DMSO-*d*_6_) *δ*_C_ (ppm): 195.28, 154.87 (d, *J* = 242.8 Hz), 153.33, 143.72, 139.75 (d, *J* = 8.4 Hz), 129.92, 129.37, 124.64 (d, *J* = 3.0 Hz), 122.08 (d, *J* = 7.6 Hz), 119.16 (d, *J* = 2.3 Hz), 117.14, 115.74 (d, *J* = 20.5 Hz), 65.93, 63.40, 52.59, 49.98 (d, *J* = 3.1 Hz), 21.69, 10.05. HR-MS: for C_22_H_26_O_3_FN_3_ [M – H]^+^ calculated 398.18745 *m*/*z*, found 398.18917 *m*/*z*.

*Butyl {4-[(4-(2-fluorophenyl)piperazin-1-yl)acetyl]phenyl}carbamate* (**6h**). Yield 2.23 g (96%); *M*_r_ 413.50; Mp 154–156 °C; ^1^H-NMR (DMSO-*d*_6_) *δ*_H_ (ppm): 10.06 (s, 1H, NHCO), 7.96 (d, 2H, Ar–H, *J* = 8.4 Hz), 7.59 (d, 2H, Ar–H, *J* = 8.4 Hz), 7.16–6.91 (m, 4H, Ar–H), 4.10 (t, 2H, CH_2_CH_2_CH_2_CH_3_, *J* = 6.6 Hz), 3.82 (s, 2H, COCH_2_N), 3.05–2.95 (m, 4H, 3,5-piperazine), 2.75–2.60 (m, 4H, 2,6-piperazine), 1.69–1.55 (m, 2H, CH_2_CH_2_CH_2_CH_3_), 1.46–1.30 (m, 2H, CH_2_CH_2_CH_2_CH_3_), 0.91 (t, 3H, CH_2_CH_2_CH_2_CH_3_, *J* = 7.3 Hz); ^13^C-NMR (DMSO-*d*_6_) *δ*_C_ (ppm): 195.33, 154.90 (d, *J* = 242.8 Hz), 153.36, 143.74, 139.78 (d, *J* = 8.4 Hz), 129.95, 129.40, 124.67 (d, *J* = 3.0 Hz), 122.13 (d, *J* = 7.6 Hz), 119.17 (d, *J* = 2.3 Hz), 117.17, 115.77 (d, *J* = 20.5 Hz), 64.16, 63.42, 52.61, 50.00 (d, *J* = 3.1 Hz), 30.39, 18.46, 13.40. HR-MS: for C_23_H_28_O_3_FN_3_ [M – H]^+^ calculated 412.20310 *m*/*z*, found 412.20490 *m*/*z*.

#### 3.2.4. General Procedure For the Preparation of Alkyl {3-/4-[2-(4-(2-Fluorophenyl)piperazin-1-yl)-1-hydroxyethyl]phenyl}carbamates (7a–h)

The synthesized alkyl {3-/4-[(4-(2-fluorophenyl)piperazin-1-yl)acetyl]phenyl}carbamates, i.e., **6a**, **6e** (1.49 g, 4.0 mmol), **6b**, **6f** (1.54 g, 4.0 mmol), **6c**, **6g** (1.60 g, 4.0 mmol), **6d** or **6h** (1.45 g, 4.0 mmol), were dissolved in hot methanol (50 mL) and solid NaBH_4_ (0.30 g, 8.0 mmol) was added in small portions [32]. The mixtures were refluxed for 1 h. When the reaction was completed (TLC control), the solvent was evaporated in vacuo, residua were treated with 100 mL of distilled water and 100 mL of chloroform. The organic layer was washed with distilled water, dried over anhydrous sodium sulphate and evaporated *in vacuo* to give crude solid products **7a**–**h**, which were crystallized from acetone. Full characterization parameters for the compounds **7a**–**h** (Scheme 2), isolated as colourless solids, are given below.

*Methyl {3-[2-(4-(2-fluorophenyl)piperazin-1-yl)-1-hydroxyethyl]phenyl}carbamate* (**7a**). Yield 1.20 g (83%); *M*_r_ 373.41; Mp 103–105 °C; IR (ATR, cm^−1^): 3382 (*υ* NH), 2951 (*υ*_as_ CH_2_), 2816 (*υ*_s_ CH_2_), 1726 (*υ* C = O), 1553 (*δ* NH), 1497 (*υ* CN), 1229 (*υ*_as_ COC), 1079 (*υ*_s_ CO), 1020 (*δ*_ip_ = C–H), 850 (*δ*_oop_ = C–H); ^1^H-NMR (DMSO-*d*_6_) *δ*_H_ (ppm): 9.61 (s, 1H, NHCO), 7.48 (s, 1H, Ar–H), 7.35 (d, 1H, Ar–H, *J* = 8.1 Hz), 7.22 (t, 1H, Ar–H, *J* = 8.1 Hz), 7.16–6.90 (m, 5H, Ar–H), 5.06 (d, 1H, CHOH, *J* = 3.7 Hz), 4.73–4.65 (m, 1H, OH), 3.66 (s, 3H, COOCH_3_), 3.10–2.95 (m, 4H, 3,5-piperazine), 2.75–2.60 (m, 4H, 2,6-piperazine), 2.55–2.38 (m, 2H, CH(OH)CH_2_N); ^13^C-NMR (DMSO-*d*_6_) *δ*_C_ (ppm): 154.83 (d, *J* = 242.8 Hz), 153.88, 145.20, 139.80 (d, *J* = 8.4 Hz), 138.73, 128.05, 124.61 (d, *J* = 3.0 Hz), 121.97 (d, *J* = 7.6 Hz), 120.08, 119.02 (d, *J* = 2.3 Hz), 116.76, 116.00, 115.72 (d, *J* = 20.5 Hz), 69.76, 66.10, 53.02, 51.34, 50.00 (d, *J* = 3.1 Hz); ESI-MS: for C_20_H_24_O_3_FN_3_ [M + H]^+^ calculated 373.42138 *m*/*z*, found 373.42095 *m*/*z*.

*Ethyl {3-[2-(4-(2-fluorophenyl)piperazin-1-yl)-1-hydroxyethyl]phenyl}carbamate* (**7b**). Yield 1.30 g (82%); *M*_r_ 387.46; Mp 118–120 °C; IR (ATR, cm^−1^): 3463 (*υ* NH), 2937 (*υ*_as_ CH_2_), 2833 (*υ*_s_ CH_2_), 1703 (*υ* C = O), 1548 (*δ* NH), 1498 (*υ* CN), 1240 (*υ*_as_ COC), 1082 (*υ*_s_ CO), 1018 (*δ*_ip_ = C–H), 852 (*δ*_oop_ = C–H); ^1^H-NMR (DMSO-*d*_6_) *δ*_H_ (ppm): 9.57 (s, 1H, NHCO), 7.50 (s, 1H, Ar–H), 7.34 (d, 1H, Ar–H, *J* = 8.1 Hz), 7.21 (t, 1H, Ar–H, *J* = 8.1 Hz), 7.13–6.91 (m, 5H, Ar–H), 5.05 (d, 1H, CHOH, *J* = 3.7 Hz), 4.73–4.65 (m, 1H, OH), 4.12 (q, 2H, CH_2_CH_3_, *J* = 7.1 Hz), 3.10–2.95 (m, 4H, 3,5-piperazine), 2.75–2.60 (m, 4H, 2,6-piperazine), 2.52–2.37 (m, 2H, CH(OH)CH_2_N), 1.24 (t, 3H, CH_2_CH_3_, *J* = 7.0 Hz ); ^13^C-NMR (DMSO-*d*_6_) *δ*_C_ (ppm): 154.83 (d, *J* = 242.8 Hz), 153.44, 145.18, 139.82 (d, *J* = 8.4 Hz), 138.82, 128.04, 124.64 (d, *J* = 3.0 Hz), 121.98 (d, *J* = 7.6 Hz), 120.01, 119.02 (d, *J* = 2.3 Hz), 116.78, 115.99, 115.76 (d, *J* = 20.5 Hz), 69.79, 66.11, 59.86, 53.03, 50.01 (d, *J* = 3.1 Hz), 14.39; ESI-MS: for C_21_H_26_O_3_FN_3_ [M + H]^+^ calculated 387.44796 *m*/*z*, found 387.44805 *m*/*z*.

*Propyl {3-[2-(4-(2-fluorophenyl)piperazin-1-yl)-1-hydroxyethyl]phenyl}carbamate* (**7c**). Yield 1.30 g (80%); *M*_r_ 401.49; Mp 103–105 °C; IR (ATR, cm^−1^): 3262 (*υ* NH), 2942 (*υ*_as_ CH_2_), 2829 (*υ*_s_ CH_2_), 1726 (*υ* C = O), 1545 (*δ* NH), 1497 (*υ* CN), 1226 (*υ*_as_ COC), 1081 (*υ*_s_ CO), 1025 (*δ*_ip_ = C–H), 848 (*δ*_oop_ = C–H); ^1^H-NMR (DMSO-*d*_6_) *δ*_H_ (ppm): 9.58 (s, 1H, NHCO), 7.51 (s, 1H, Ar–H), 7.34 (d, 1H, Ar–H, *J* = 8.1 Hz), 7.21 (t, 1H, Ar–H, *J* = 8.1 Hz), 7.13–6.91 (m, 5H, Ar–H), 5.05 (d, 1H, CHOH, *J* = 3.7 Hz), 4.73–4.65 (m, 1H, OH), 4.03 (t, 2H, CH_2_CH_2_CH_3_, *J* = 6.6 Hz), 3.07–2.93 (m, 4H, 3,5-piperazine), 2.73–2.59 (m, 4H, 2,6-piperazine), 2.55–2.38 (m, 2H, CH(OH)CH_2_N), 1.73–1.55 (m, 2H, CH_2_CH_2_CH_3_), 0.93 (t, 3H, CH_2_CH_2_CH_3_, *J* = 7.3 Hz); ^13^C-NMR (DMSO-*d*_6_) *δ*_C_ (ppm): 154.83 (d, *J* = 242.8 Hz), 153.57, 145.23, 139.85 (d, *J* = 8.4 Hz), 138.87, 128.08, 124.67 (d, *J* = 3.0 Hz), 122.03 (d, *J* = 7.6 Hz), 120.04, 119.06 (d, *J* = 2.3 Hz), 116.81, 116.02, 115.77 (d, *J* = 20.5 Hz), 69.80, 66.16, 65.46, 53.07, 50.05 (d, *J* = 3.1 Hz), 21.82, 10.13; ESI-MS: for C_22_H_28_O_3_FN_3_ [M + H]^+^ calculated 401.47454 *m*/*z*, found 401.47421 *m*/*z*.

*Butyl {3-[2-(4-(2-fluorophenyl)piperazin-1-yl)-1-hydroxyethyl]phenyl}carbamate* (**7d**). Yield 1.50 g (90%); *M*_r_ 415.52; Mp 107–110 °C; IR (ATR, cm^−1^): 3294 (*υ* NH), 2940 (*υ*_as_ CH_2_), 2830 (*υ*_s_ CH_2_), 1706 (*υ* C = O), 1541 (*δ* NH), 1497 (*υ* CN), 1228 (*υ*_as_ COC), 1082 (*υ*_s_ CO), 1018 (*δ*_ip_ = C–H), 845 (*δ*_oop_ = C–H); ^1^H-NMR (DMSO-*d*_6_) *δ*_H_ (ppm): 9.57 (s, 1H, NHCO), 7.51 (s, 1H, Ar–H), 7.33 (d, 1H, Ar–H, *J* = 8.1 Hz), 7.20 (t, 1H, Ar–H, *J* = 8.1 Hz), 7.13–6.91 (m, 5H, Ar–H), 5.04 (d, 1H, CHOH, *J* = 3.7 Hz), 4.73–4.65 (m, 1H, OH), 4.07 (t, 2H, CH_2_CH_2_CH_2_CH_3_, *J* = 6.6 Hz), 3.10–2.95 (m, 4H, 3,5-piperazine), 2.75–2.60 (m, 4H, 2,6-piperazine), 2.53–2.37 (m, 2H, CH(OH)CH_2_N), 1.67–1.53 (m, 2H, CH_2_CH_2_CH_2_CH_3_), 1.47–1.29 (m, 2H, CH_2_CH_2_CH_2_CH_3_), 0.92 (t, 3H, CH_2_CH_2_CH_2_CH_3_, *J* = 7.3 Hz); ^13^C-NMR (DMSO-*d*_6_) *δ*_C_ (ppm): 154.85 (d, *J* = 242.8 Hz), 153.56, 145.21, 139.83 (d, *J* = 8.4 Hz), 138.85, 128.07, 124.65 (d, *J* = 3.0 Hz), 122.01 (d, *J* = 7.6 Hz), 120.02, 119.03 (d, *J* = 2.3 Hz), 116.78, 115.97, 115.77 (d, *J* = 20.5 Hz), 69.80, 66.14, 63.64, 53.07, 50.04 (d, *J* = 3.1 Hz), 30.52, 18.49, 13.45; ESI-MS: for C_23_H_30_O_3_FN_3_ [M + H]^+^ calculated 415.50112 *m*/*z*, found 415.50207 *m*/*z*.

*Methyl {4-[2-(4-(2-fluorophenyl)piperazin-1-yl)-1-hydroxyethyl]phenyl}carbamate* (**7e**). Yield 1.42 g (94%); *M*_r_ 373.41; Mp 143–146 °C; IR (ATR, cm^−1^): 3340 (*υ* NH), 2975 (*υ*_as_ CH_2_), 2808 (*υ*_s_ CH_2_), 1702 (*υ* C = O), 1552 (*δ* NH), 1502 (*υ* CN), 1234 (*υ*_as_ COC), 1063 (*υ*_s_ CO), 1021 (*δ*_ip_ = C–H), 855 (*δ*_oop_ = C–H); ^1^H-NMR (DMSO-*d*_6_) *δ*_H_ (ppm): 9.59 (s, 1H, NHCO), 7.38 (d, 2H, Ar–H, *J* = 8.4 Hz), 7.25 (d, 2H, Ar–H, *J* = 8.4 Hz), 7.16–6.90 (m, 4H, Ar–H), 4.96 (d, 1H, CHOH, *J* = 3.7 Hz), 4.75–4.63 (m, 1H, OH), 3.64 (s, 3H, COOCH_3_), 3.12–2.90 (m, 4H, 3,5-piperazine), 2.75–2.55 (m, 4H, 2,6-piperazine), 2.55–2.35 (m, 2H, CH(OH)CH_2_N); ^13^C-NMR (DMSO-*d*_6_) *δ*_C_ (ppm): 154.85 (d, *J* = 242.8 Hz), 153.92, 139.82 (d, *J* = 8.4 Hz), 138.55, 137.69, 126.26, 124.64 (d, *J* = 3.0 Hz), 121.98 (d, *J* = 7.6 Hz), 119.05 (d, *J* = 2.3 Hz), 117.87, 115.74 (d, *J* = 20.5 Hz), 69.36, 66.04, 53.03, 51.37, 50.05 (d, *J* = 3.1 Hz); ESI-MS: for C_20_H_24_O_3_FN_3_ [M + H]^+^ calculated 373.42138 *m*/*z*, found 373.42142 *m*/*z*.

*Ethyl {4-[2-(4-(2-fluorophenyl)piperazin-1-yl)-1-hydroxyethyl]phenyl}carbamate* (**7f**). Yield 1.40 g (88%); *M*_r_ 387.46; Mp 142–145 °C; IR (ATR, cm^−1^): 3340 (*υ* NH), 2977 (*υ*_as_ CH_2_), 2808 (*υ*_s_ CH_2_), 1711 (*υ* C = O), 1549 (*δ* NH), 1503 (*υ* CN), 1234 (*υ*_as_ COC), 1060 (*υ*_s_ CO), 1018 (*δ*_ip_ = C–H), 860 (*δ*_oop_ = C–H); ^1^H-NMR (DMSO-*d*_6_) *δ*_H_ (ppm): 9.54 (s, 1H, NHCO), 7.38 (d, 2H, Ar–H, *J* = 8.4 Hz), 7.22 (d, 2H, Ar–H, *J* = 8.4 Hz), 7.15–6.91 (m, 4H, Ar–H), 4.96 (d, 1H, CHOH, *J* = 3.7 Hz), 4.71–4.61 (m, 1H, OH), 4.08 (q, 2H, CH_2_CH_3_, *J* = 7.0 Hz), 3.06–2.91 (m, 4H, 3,5-piperazine), 2.71–2.54 (m, 4H, 2,6-piperazine), 2.52–2.34 (m, 2H, CH(OH)CH_2_N), 1.21 (t, 3H, CH_2_CH_3_, *J* = 7.0 Hz); ^13^C-NMR (DMSO-*d*_6_) *δ*_C_ (ppm): 154.85 (d, *J* = 242.8 Hz), 153.47, 139.82 (d, *J* = 8.4 Hz), 138.46, 137.76, 126.23, 124.63 (d, *J* = 3.0 Hz), 121.97 (d, *J* = 7.6 Hz), 119.03 (d, *J* = 2.3 Hz), 117.82, 115.73 (d, *J* = 20.5 Hz), 69.36, 66.05, 59.88, 53.03, 50.07 (d, *J* = 3.1 Hz), 14.39; ESI-MS: for C_21_H_26_O_3_FN_3_ [M + H]^+^ calculated 387.44796 *m*/*z*, found 387.44822 *m*/*z*.

*Propyl {4-[2-(4-(2-fluorophenyl)piperazin-1-yl)-1-hydroxyethyl]phenyl}carbamate* (**7g**). Yield 1.53 g (92%); *M*_r_ 401.49; Mp 149–152 °C; IR (ATR, cm^−1^): 3339 (*υ* NH), 2966 (*υ*_as_ CH_2_), 2808 (*υ*_s_ CH_2_), 1698 (*υ* C = O), 1550 (*δ* NH), 1505 (*υ* CN), 1235 (*υ*_as_ COC), 1073 (*υ*_s_ CO), 1015 (*δ*_ip_ = C–H), 857 (*δ*_oop_ = C–H); ^1^H-NMR (DMSO-*d*_6_) *δ*_H_ (ppm): 9.56 (s, 1H, NHCO), 7.39 (d, 2H, Ar–H, *J* = 8.4 Hz), 7.24 (d, 2H, Ar–H, *J* = 8.4 Hz), 7.16–6.89 (m, 4H, Ar–H), 4.98 (d, 1H, CHOH, *J* = 3.7 Hz), 4.73–4.61 (m, 1H, OH), 4.01 (t, 2H, CH_2_CH_2_CH_3_, *J* = 6.6 Hz), 3.08–2.91 (m, 4H, 3,5-piperazine), 2.68–2.54 (m, 4H, 2,6-piperazine), 2.54–2.33 (m, 2H, CH(OH)CH_2_N), 1.71–1.52 (m, 2H, CH_2_CH_2_CH_3_), 0.92 (t, 3H, CH_2_CH_2_CH_3_, *J* = 7.3 Hz); ^13^C-NMR (DMSO-*d*_6_) *δ*_C_ (ppm): 154.86 (d, *J* = 242.8 Hz), 153.60, 139.82 (d, *J* = 8.4 Hz), 138.46, 137.79, 126.24, 124.64 (d, *J* = 3.0 Hz), 122.00 (d, *J* = 7.6 Hz), 119.05 (d, *J* = 2.3 Hz), 117.84, 115.75 (d, *J* = 20.5 Hz), 69.38, 66.05, 65.48, 53.05, 50.06 (d, *J* = 3.1 Hz), 21.79, 10.11; ESI-MS: for C_22_H_28_O_3_FN_3_ [M + H]^+^ calculated 401.47454 *m*/*z*, found 401.47510 *m*/*z*.

*Butyl {4-[2-(4-(2-fluorophenyl)piperazin-1-yl)-1-hydroxyethyl]phenyl}carbamate* (**7h**). Yield 1.62 g (94%); *M*_r_ 415.52; Mp 140–143 °C; IR (ATR, cm^−1^): 3336 (*υ* NH), 2954 (*υ*_as_ CH_2_), 2808 (*υ*_s_ CH_2_), 1698 (*υ* C = O), 1549 (*δ* NH), 1527 (*υ* CN), 1228 (*υ*_as_ COC), 1076 (*υ*_s_ CO), 1018 (*δ*_ip_ = C–H), 852 (*δ*_oop_ = C–H); ^1^H-NMR (DMSO-*d*_6_) *δ*_H_ (ppm): 9.56 (s, 1H, NHCO), 7.40 (d, 2H, Ar–H, *J* = 8.4 Hz), 7.25 (d, 2H, Ar–H, *J* = 8.4 Hz), 7.16–6.89 (m, 4H, Ar–H), 4.99 (d, 1H, CHOH, *J* = 3.7 Hz), 4.72–4.59 (m, 1H, OH), 4.06 (t, 2H, CH_2_CH_2_CH_2_CH_3_, *J* = 6.6 Hz), 3.09–2.94 (m, 4H, 3,5-piperazine), 2.64–2.55 (m, 4H, 2,6-piperazine), 2.55–2.36 (m, 2H, CH(OH)CH_2_N), 1.65–1.52 (m, 2H, CH_2_CH_2_CH_2_CH_3_), 1.46–1.28 (m, 2H, CH_2_CH_2_CH_2_CH_3_), 0.91 (t, 3H, CH_2_CH_2_CH_2_CH_3_, *J* = 7.3 Hz); ^13^C-NMR (DMSO-*d*_6_) *δ*_C_ (ppm): 154.87 (d, *J* = 242.8 Hz), 153.62, 139.84 (d, *J* = 8.4 Hz), 138.47, 137.81, 126.26, 124.66 (d, *J* = 3.0 Hz), 122.02 (d, *J* = 7.6 Hz), 119.07 (d, *J* = 2.3 Hz), 117.86, 115.76 (d, *J* = 20.5 Hz), 69.38, 66.07, 63.67, 53.05, 50.06 (d, *J* = 3.1 Hz), 30.52, 18.49, 13.43; ESI-MS: for C_23_H_30_O_3_FN_3_ [M + H]^+^ calculated 415.50112 *m*/*z*, found 415.50154 *m*/*z*.

#### 3.2.5. General Procedure For the Preparation of 1-(2-{3-/4-[(Alkoxycarbonyl)amino]phenyl}-2-hydroxyethyl)-4-(2-fluorophenyl)piperazin-1-ium Chlorides (**8a**–**h**)

The solution of a particular alkyl {3-/4-[2-(4-(2-fluorophenyl)piperazin-1-yl)-1-hydroxyeth-yl]phenyl}carbamate, i.e, **7a**, **7e** (0.71 g, 1.9 mmol), **7b**, **7f** (0.74 g, 1.9 mmol), **7c**, **7g** (0.76 g, 1.9 mmol), **7d** or **7h** (0.79 g, 1.9 mmol), in 40 mL of chloroform was treated with a saturated solution of hydrogen chloride in diethyl ether and stirred for 5 h at laboratory temperature. The solvents were removed *in vacuo* and solid crude products **8a**–**h** were crystallized from acetone. Full characterization parameters for the target compounds **8a**–**h** (Scheme 2), isolated as colourless solids, are provided below.

*1-(2-Hydroxy-2-{3-[(methoxycarbonyl)amino]phenyl}ethyl)-4-(2-fluorophenyl)piperazin-1-ium chloride* (**8a**). Yield 0.75 g (88%); *M*_r_ 409.89; Mp 139–141 °C; *R*_f_ 0.40; IR (ATR, cm^−1^): 3266 (*υ* NH), 2952 (*υ*_as_ CH_2_), 2815 (*υ*_s_ CH_2_), 1718 (*υ* C = O), 1612 (*υ* C = C), 1552 (*δ* NH), 1491 (*υ* CN), 1233 (*υ*_as_ COC), 1081 (*υ*_s_ CO), 1020 (*δ*_ip_ = C–H), 848 (*δ*_oop_ = C–H); ^1^H-NMR (DMSO-*d*_6_) *δ*_H_ (ppm): 10.71 (m, 1H, NH^+^), 9.60 (s, 1H, NHCO), 7.51 (s, 1H, Ar–H), 7.44 (t, 1H, *J* = 8.0 Hz), 7.35 (d, 1H, Ar–H, *J* = 8.0 Hz), 7.25 (t, 1H, Ar–H, *J* = 8.0 Hz), 7.30–7.15 (m, 5H, Ar–H), 5.25 (dd, 1H, CHOH, *J* = 4.1 Hz, *J* = 9.0 Hz), 4.70–4.65 (m, 1H, OH), 3.65 (s, 3H, COOCH_3_), 3.65–3.55 (m, 4H, 3,5-piperazine), 4.10–3.25 (m, 6H, CH(OH)CH_2_N + 2,6-piperazine); ^13^C-NMR (DMSO-*d*_6_) *δ*_C_ (ppm): 153.25 (d, *J* = 249.1 Hz), 152.91, 144.68, 139.37 (d, *J* = 8.3 Hz), 137.21, 128.16, 124.35 (d, *J* = 3.1 Hz), 121.42 (d, *J* = 7.6 Hz), 120.15, 118.46 (d, *J* = 2.2 Hz), 116.24, 116.07, 115.11 (d, *J* = 20.4 Hz), 69.55, 66.14, 53.34, 51.26, 50.07 (d, *J* = 3.0 Hz); ESI-MS: for C_20_H_25_O_3_FN_3_ [M + H]^+^ calculated 374.42932 *m*/*z*, found 374.42873 *m*/*z*. Anal. Calcd. for C_20_H_25_O_3_ClFN_3_ (409.89): C, 58.61%; H, 6.15%; N, 10.25%. Found: C, 58.65%; H, 6.18%; N, 10.15%.

*1-(2-{3-[(Ethoxycarbonyl)amino]phenyl}-2-hydroxyethyl)-4-(2-fluorophenyl)piperazin-1-ium chloride* (**8b**). Yield 0.80 g (91%); *M*_r_ 423.91; Mp 117–119 °C; *R*_f_ 0.53; IR (ATR, cm^−1^): 3425 (*υ* NH), 2983 (*υ*_as_ CH_2_), 2852 (*υ*_s_ CH_2_), 1718 (*υ* C = O), 1610 (*υ* C = C), 1547 (*δ* NH), 1496 (*υ* CN), 1229 (*υ*_as_ COC), 1068 (*υ*_s_ CO), 1018 (*δ*_ip_ = C–H), 852 (*δ*_oop_ = C–H); ^1^H-NMR (DMSO-*d*_6_) *δ*_H_ (ppm): 10.73 (m, 1H, NH^+^), 9.57 (s, 1H, NHCO), 7.50 (s, 1H, Ar–H), 7.45 (t, 1H, Ar–H, *J* = 8.0 Hz), 7.35 (d, 1H, Ar–H, *J* = 8.1 Hz), 7.27–7.15 (m, 5H, Ar–H), 5.24 (dd, 1H, CHOH, *J* = 4.1 Hz, *J* = 9.0 Hz), 4.73–4.65 (m, 1H, OH), 4.20 (q, 2H, CH_2_CH_3_, *J* = 7.0 Hz), 3.60–3.50 (m, 4H, 3,5-piperazine), 4.00–3.25 (m, 6H, CH(OH)CH_2_N + 2,6-piperazine), 1.30 (t, 3H, CH_2_CH_3_, *J* = 7.0 Hz); ^13^C-NMR (DMSO-*d*_6_) *δ*_C_ (ppm): 154.81 (d, *J* = 241.4 Hz), 153.47, 146.78, 139.94 (d, *J* = 8.4 Hz), 138.57, 128.16, 124.02 (d, *J* = 3.0 Hz), 121.64 (d, *J* = 7.5 Hz), 119.78, 119.07 (d, *J* = 2.3 Hz), 116.35, 116.28, 115.01 (d, *J* = 20.4 Hz), 69.74, 65.89, 59.34, 53.07, 50.12 (d, *J* = 3.1 Hz), 14.21; ESI-MS: for C_21_H_27_O_3_FN_3_ [M + H]^+^ calculated 388.45590 *m*/*z*, found 388.45615 *m*/*z*. Anal. Calcd. for C_21_H_27_O_3_ClFN_3_ (423.91): C, 59.50%; H, 6.42%; N, 9.91%. Found: C, 59.33%; H, 6.31%; N, 10.06%.

*1-(2-Hydroxy-2-{3-[(propoxycarbonyl)amino]phenyl}ethyl)-4-(2-fluorophenyl)piperazin-1-ium chloride* (**8c**). Yield 0.82 g (91%); *M*_r_ 437.94; Mp 99–101 °C; *R*_f_ 0.66; IR (ATR, cm^−1^): 3402 (*υ* NH), 2964 (*υ*_as_ CH_2_), 2837 (*υ*_s_ CH_2_), 1724 (*υ* C = O), 1612 (*υ* C = C), 1546 (*δ* NH), 1498 (*υ* CN), 1224 (*υ*_as_ COC), 1072 (*υ*_s_ CO), 1022 (*δ*_ip_ = C–H), 850 (*δ*_oop_ = C–H); ^1^H-NMR (DMSO-*d*_6_) *δ*_H_ (ppm): 10.78 (m, 1H, NH^+^), 9.58 (s, 1H, NHCO), 7.50 (s, 1H, Ar–H), 7.44 (t, 1H, Ar–H, *J* = 8.0 Hz), 7.35 (d, 1H, Ar–H, *J* = 8.0 Hz), 7.20 (t, 1H, Ar–H, *J* = 8.1 Hz), 7.30–7.18 (m, 5H, Ar–H), 5.24 (dd, 1H, CHOH, *J* = 4.0 Hz, *J* = 9.0 Hz), 4.73–4.65 (m, 1H, OH), 4.12 (t, 2H, CH_2_CH_2_CH_3_, *J* = 7.0 Hz), 3.60–3.45 (m, 4H, 3,5-piperazine), 4.00–3.42 (m, 6H, CH(OH)CH_2_N + 2,6-piperazine), 1.69–1.57 (m, 2H, CH_2_CH_2_CH_3_), 0.95 (t, 3H, CH_2_CH_2_CH_3_, *J* = 7.0 Hz); ^13^C-NMR (DMSO-*d*_6_) *δ*_C_ (ppm): 154.80 (d, *J* = 242.7 Hz), 153.78, 145.15, 138.19 (d, *J* = 8.4 Hz), 137.45, 128.49, 124.02 (d, *J* = 3.0 Hz), 122.09 (d, *J* = 7.7 Hz), 119.97, 119.03 (d, *J* = 2.3 Hz), 116.76, 116.08, 115.29 (d, *J* = 20.5 Hz), 69.54, 66.17, 65.76, 53.27, 50.03 (d, *J* = 3.1 Hz), 21.81, 10.15; ESI-MS: for C_22_H_29_O_3_FN_3_ [M + H]^+^ calculated 402.48248 *m*/*z*, found 402.48197 *m*/*z*. Anal. Calcd. for C_22_H_29_O_3_ClFN_3_ (437.94): C, 60.34%; H, 6.67%; N, 9.59%. Found: C, 60.12%; H, 6.81%; N, 9.37%.

*1-(2-{3-[(Butoxycarbonyl)amino]phenyl}-2-hydroxyethyl)-4-(2-fluorophenyl)piperazin-1-ium chloride* (**8d**). Yield 0.88 g (95%); *M*_r_ 451.97; Mp 98–100 °C; *R*_f_ 0.73; IR (ATR, cm^−1^): 3416 (*υ* NH), 2959 (*υ*_as_ CH_2_), 2837 (*υ*_s_ CH_2_), 1730 (*υ* C = O), 1604 (*υ* C = C), 1543 (*δ* NH), 1495 (*υ* CN), 1221 (*υ*_as_ COC), 1074 (*υ*_s_ CO), 1016 (*δ*_ip_ = C–H), 840 (*δ*_oop_ = C–H); ^1^H-NMR (DMSO-*d*_6_) *δ*_H_ (ppm): 10.78 (m, 1H, NH^+^), 9.57 (s, 1H, NHCO), 7.50 (s, 1H, Ar–H), 7.44 (t, 1H, Ar–H, *J* = 8.1 Hz), 7.34 (d, 1H, Ar–H, *J* = 8.1 Hz), 7.25–7.18 (m, 5H, Ar–H), 5.25 (dd, 1H, CHOH, *J* = 4.0 Hz, *J* = 9.0 Hz), 4.73–4.65 (m, 1H, OH), 4.18 (t, 2H, CH_2_CH_2_CH_2_CH_3_, *J* = 7.0 Hz), 3.60–3.45 (m, 4H, 3,5-piperazine), 4.00–3.40 (m, 6H, CH(OH)CH_2_N + 2,6-piperazine), 1.67–1.58 (m, 2H, CH_2_CH_2_CH_2_CH_3_, *J* = 7.0), 1.40–1.30 (m, 2H, CH_2_CH_2_CH_2_CH_3_), 0.93 (t, 3H, CH_2_CH_2_CH_2_CH_3_, *J* = 7.0 Hz); ^13^C-NMR (DMSO-*d*_6_) *δ*_C_ (ppm): 154.90 (d, *J* = 241.4 Hz), 153.13, 145.61, 139.70 (d, *J* = 8.5 Hz), 138.62, 128.17, 124.34 (d, *J* = 3.1 Hz), 122.14 (d, *J* = 7.6 Hz), 120.07, 119.26 (d, *J* = 2.3 Hz), 116.35, 115.82, 115.61 (d, *J* = 20.4 Hz), 69.81, 66.38, 63.94, 53.23, 50.17 (d, *J* = 3.1 Hz), 30.60, 18.26, 13.33; ESI-MS: for C_23_H_31_O_3_FN_3_ [M + H]^+^ calculated 416.50906 *m*/*z*, found 416.50915 *m*/*z*. Anal. Calcd. for C_23_H_31_O_3_ClFN_3_ (451.97): C, 61.12%; H, 6.91%; N, 9.30%. Found: C, 61.02%; H, 6.85%; N, 9.42%.

*1-(2-Hydroxy-2-{4-[(methoxycarbonyl)amino]phenyl}ethyl)-4-(2-fluorophenyl)piperazin-1-ium chloride* (**8e**). Yield 0.78 g (84%); *M*_r_ 409.89; Mp 218–220 °C; *R*_f_ 0.31; IR (ATR, cm^−1^): 3377 (*υ* NH), 2963 (*υ*_as_ CH_2_), 2831 (*υ*_s_ CH_2_), 1719 (*υ* C = O), 1610 (*υ* C = C), 1550 (*δ* NH), 1487 (*υ* CN), 1234 (*υ*_as_ COC), 1073 (*υ*_s_ CO), 1020 (*δ*_ip_ = C–H), 854 (*δ*_oop_ = C–H); ^1^H-NMR (DMSO-*d*_6_) *δ*_H_ (ppm): 10.42 (s, 1H, NH^+^), 9.71 (s, 1H, NHCO), 7.47 (d, 2H, Ar–H, *J* = 8.5 Hz), 7.32 (d, 2H, Ar–H, *J* = 8.5 Hz), 7.23–7.00 (m, 4H, Ar–H), 6.20 (s, 1H, OH), 5.14 (dd, 1H, CHOH, *J* = 4.8 Hz, *J* = 7.8 Hz), 3.66 (s, 3H, COOCH_3_), 3.75–3.11 (m, 10H, CH(OH)CH_2_N + piperazine); ^13^C-NMR (DMSO-*d*_6_) *δ*_C_ (ppm): 154.73 (d, *J* = 244.6 Hz), 153.91, 138.62, 138.25 (d, *J* = 8.6 Hz), 135.32, 126.41, 124.94 (d, *J* = 3.1 Hz), 123.27 (d, *J* = 7.8 Hz), 119.51 (d, *J* = 2.2 Hz), 117.93, 116.10 (d, *J* = 20.3 Hz), 66.15, 61.59, 52.32, 51.54, 50.30, 46.82, 46.51; ESI-MS: for C_20_H_25_O_3_FN_3_ [M + H]^+^ calculated 374.42932 *m*/*z*, found 374.42951 *m*/*z*. Anal. Calcd. for C_20_H_25_O_3_ClFN_3_ (409.89): C, 58.61%; H, 6.15%; N, 10.25%. Found: C, 58.62%; H, 6.31%; N, 10.06%.

*1-(2-{4-[(Ethoxycarbonyl)amino]phenyl}-2-hydroxyethyl)-4-(2-fluorophenyl)piperazin-1-ium chloride* (**8f**). Yield 0.79 g (91%); M_r_ 423.91; Mp 208–210 °C; *R*_f_ 0.45; IR (ATR, cm^−1^): 3354 (*υ* NH), 2980 (*υ*_as_ CH_2_), 2851 (*υ*_s_ CH_2_), 1719 (*υ* C = O), 1600 (*υ* C = C), 1552 (*δ* NH), 1519 (*υ* CN), 1232 (*υ*_as_ COC), 1079 (*υ*_s_ CO), 1016 (*δ*_ip_ = C–H), 858 (*δ*_oop_ = C–H); ^1^H-NMR (DMSO-*d*_6_) *δ*_H_ (ppm): 10.46 (m, 1H, NH^+^), 9.67 (s, 1H, NHCO), 7.47 (d, 2H, Ar–H, *J* = 8.5 Hz), 7.32 (d, 2H, Ar–H, *J* = 8.5 Hz), 7.25–7.00 (m, 4H, Ar–H), 6.21 (s, 1H, OH), 5.14 (dd, 1H, CHOH, OH, *J* = 4.8 Hz, *J* = 7.8 Hz), 4.12 (q, 2H, CH_2_CH_3_, *J* = 7.0 Hz), 3.78–3.14 (m, 10H, CH(OH)CH_2_N + piperazine), 1.24 (t, 3H, CH_2_CH_3_, *J* = 7.0 Hz); ^13^C-NMR (DMSO-*d*_6_) *δ*_C_ (ppm): 154.74 (d, *J* = 244.4 Hz), 153.36, 138.78, 138.21 (d, *J* = 8.7 Hz), 135.20, 126.34, 124.89 (d, *J* = 3.3 Hz), 123.31 (d, *J* = 8.0 Hz), 119.45 (d, *J* = 2.2 Hz), 117.91, 116.11 (d, *J* = 20.5 Hz), 66.33, 61.74, 60.56, 52.34, 50.30, 46.76, 46.64, 14.38; ESI-MS: for C_21_H_27_O_3_FN_3_ [M + H]^+^ calculated 388.45590 *m*/*z*, found 388.45541 *m*/*z*. Anal. Calcd. for C_21_H_27_O_3_ClFN_3_ (423.91): C, 59.50%; H, 6.42%; N, 9.91%. Found: C, 59.45%; H, 6.39%; N, 10.02%.

*1-(2-Hydroxy-2-{4-[(propoxycarbonyl)amino]phenyl}ethyl)-4-(2-fluorophenyl)piperazin-1-ium chloride* (**8g**). Yield 0.77 g (86%); *M*_r_ 437.94; Mp 212–214 °C; *R*_f_ 0.50; IR (ATR, cm^−1^): 3392 (*υ* NH), 2973 (*υ*_as_ CH_2_), 2847 (*υ*_s_ CH_2_), 1720 (*υ* C = O), 1605 (*υ* C = C), 1543 (*δ* NH), 1501 (*υ* CN), 1231 (*υ*_as_ COC), 1079 (*υ*_s_ CO), 1012 (*δ*_ip_ = C–H), 855 (*δ*_oop_ = C–H); ^1^H-NMR (DMSO-*d*_6_) *δ*_H_ (ppm): 10.56 (m, 1H, NH^+^), 9.68 (s, 1H, NHCO), 7.48 (d, 2H, Ar–H, *J* = 8.5 Hz), 7.33 (d, 2H, Ar–H, *J* = 8.5 Hz), 7.23–7.00 (m, 4H, Ar–H), 6.20 (s, 1H, OH), 5.15 (dd, 1H, CHOH, *J* = 4.8 Hz, *J* = 7.9 Hz), 4.03 (t, 2H, CH_2_CH_2_CH_3_, *J* = 6.8 Hz), 3.79–3.16 (m, 10H, CH(OH)CH_2_N + piperazine), 1.63 (m, 2H, CH_2_CH_2_CH_3_, *J* = 7.1 Hz), 0.93 (t, 3H, CH_2_CH_2_CH_3_, *J* = 7.3 Hz); ^13^C-NMR (DMSO-*d*_6_) *δ*_C_ (ppm): 154.74 (d, *J* = 244.7 Hz), 153.45, 138.78, 138.21 (d, *J* = 8.7 Hz), 135.32, 126.42, 124.89 (d, *J* = 3.3 Hz), 123.31 (d, *J* = 7.9 Hz), 119.45 (d, *J* = 2.2 Hz), 117.91, 116.10 (d, *J* = 20.2 Hz), 66.29, 65.56, 61.77, 52.23, 50.42, 46.80, 46.64, 21.78, 10.21; ESI-MS: for C_22_H_29_O_3_FN_3_ [M + H]^+^ calculated 402.48248 *m*/*z*, found 402.48217 *m*/*z*. Anal. Calcd. for C_22_H_29_O_3_ClFN_3_ (437.94): C, 60.34%; H, 6.67%; N, 9.59%. Found: C, 60.21%; H, 6.75%; N, 9.43%.

*1-(2-{4-[(Butoxycarbonyl)amino]phenyl}-2-hydroxyethyl)-4-(2-fluorophenyl)piperazin-1-ium chloride* (**8h**). Yield 0.89 g (96%); *M*_r_ 451.97; Mp 219–220 °C; *R*_f_ 0.56; IR (ATR, cm^−1^): 3361 (*υ* NH), 2957 (*υ*_as_ CH_2_), 2825 (*υ*_s_ CH_2_), 1722 (*υ* C = O), 1610 (*υ* C = C), 1546 (*δ* NH), 1499 (*υ* CN), 1232 (*υ*_as_ COC), 1075 (*υ*_s_ CO), 1020 (*δ*_ip_ = C–H), 852 (*δ*_oop_ = C–H); ^1^H-NMR (DMSO-*d*_6_) *δ*_H_ (ppm): 10.46 (m, 1H, NH^+^), 9.67 (s, 1H, NHCO), 7.48 (d, 2H, Ar–H, *J* = 8.5 Hz), 7.33 (d, 2H, Ar–H, *J* = 8.5 Hz), 7.24–7.01 (m, 4H, Ar–H), 6.22 (s, 1H, OH), 5.14 (dd, 1H, CHOH, *J* = 4.8 Hz, *J* = 7.8 Hz), 4.07 (t, 2H, CH_2_CH_2_CH_2_CH_3_, *J* = 6.6 Hz), 3.78–3.16 (m, 10H, CH(OH)CH_2_N + piperazine), 1.62 (q, 2H, CH_2_CH_2_CH_2_CH_3_, *J* = 7.0 Hz), 1.38 (m, 2H, CH_2_CH_2_CH_2_CH_3_, *J* = 7.4 Hz), 0.92 (t, 3H, CH_2_CH_2_CH_2_CH_3_, *J* = 7.3 Hz); ^13^C-NMR (DMSO-*d*_6_) *δ*_C_ (ppm): 154.74 (d, *J* = 244.4 Hz), 153.52, 138.81, 138.23 (d, *J* = 8.6 Hz), 135.35, 126.41, 124.89 (d, *J* = 3.1 Hz), 123.33 (d, *J* = 7.3 Hz), 119.50 (d, *J* = 2.2 Hz), 117.92, 116.10 (d, *J* = 20.2 Hz), 66.29, 63.78, 61.74, 52.34, 50.32, 46.82, 46.61, 30.52, 18.48, 13.47; ESI-MS: for C_23_H_31_O_3_FN_3_ [M + H]^+^ calculated 416.50906 *m*/*z*, found 416.50934 *m*/*z*. Anal. Calcd. for C_23_H_31_O_3_ClFN_3_ (451.97): C, 61.12%; H, 6.91%; N, 9.30%. Found: C, 61.05%; H, 6.90%; N, 9.37%.

### 3.3. Lipophilicity Parameter Determination

Lipohydrophilic properties of the final compounds **8a**–**h** were characterized by the *R*_M_ (RP-TLC), log *k* and log *k*_w_ (RP-HPLC) parameters, respectively.

#### 3.3.1. Reversed-Phase Thin-Layer Chromatography (RP-TLC)

The *R*_M_ values were calculated according to Equation (5) from the *R*_f_ parameters observed in a *S*_M_ mobile phase: hydrochloric acid (*c* = 1.0 M)/acetone (4:1, *v*/*v*):

*R*_M_ = log (1/*R*_f_ – 1) (5)

For the experiments, aluminium sheets pre-coated with silica gel 60 F_254_ (0.25 mm thickness; Merck) were impregnated by a variously concentrated silicone oil in heptane, which ranged from 1% to 5%. The plates were separately spotted with 2 µL of methanolic solutions of each compound (*c* = 1 mg/mL), starting points were 1 cm from a bottom edge of these plates. The chromatographic plates were developed in glass developing chambers saturated for 30 min by the *S*_M_ mobile phase. Development was carried out upon 15 cm from a starting line by an ascending technique [73,74]. After being developed, the plates were dried at room temperature. Detection of zones was performed under iodine vapours/UV light at *λ* = 254 nm. Each experiment was run in triplicate at 21 °C, the *R*_M_ values of analytes were calculated separately for each run.

Optimal differences in *R*_M_ values within both homological groups **8a**–**d** and **8e**–**h** were observed if 1% silicone oil in heptane was chosen. The *R*_f_s were found in a range from 0.19 (**8h**) to 0.78 (**8a**; Table 1). All the calculated average *R*_M_ values were summarized in Table S1 (Supplementary Materials).

#### 3.3.2. Reversed-Phase High-Performance Liquid Chromatography (RP-HPLC)

Methanol (MeOH)/water mobile phases with a various volume ratio of the organic modifier and water (60:40, 70:30, 80:20 and 85:15, respectively; *v*/*v*) were chosen. A concentration of all analyzed compounds **8a**–**h** was *c* = 0.25 mg/mL. An queous solution of potassium nitrate (*c* = 0.1 M) was used for the determination of dead time *t*_0_ = 1.295 min. The capacity (retention) factor *k* values (Table 1) were calculated by Equation (6) as follows:

*k* = (*t*_r_ – *t*_0_)/*t*_0_ (6)

where the *t*_r_ and *t*_0_ parameters were the retention times of a solute (*t*_r_) and unretained compound (potassium nitrate; *t*_0_), respectively. The observed retention (*t*_r_) and dead (*t*_0_) times were means of three independent determinations [73,74].

The log *k*_w_ values, i.e., the logarithms of extrapolated capacity (retention) factors for 100% water in the isocratic RP-HPLC, were determined from intercepts of linear plots between the log *k* and *ϕ*_M_ (a volume fraction of an organic modifier in the isocratic elution RP-HPLC) according to Equation (7):

log *k* = log *k*_w_ – *S* × *ϕ*_M_ (7)

where the *S* parameter represented the slope of a regression curve, which was related to the solvent strength of a pure organic solvent [46,75].

### 3.4. Electronic Properties Determination

The log *ε* values characterizing methanolic solutions of the analyzed compounds **8a**–**h** (*c* = 3.0 × 10^−5^ M) were estimated at *λ*_1_ = 208–210 nm, *λ*_2(Ch-T)_ = 238–240 nm and *λ*_3_ = 274–276 nm (Table 3), respectively, in a near ultraviolet (quarz) region of the electromagnetic spectrum between 200 and 400 nm [47]. The log *ε* values for the observed absorption maxima were calculated according to the Lambert-Beer´s law discussed in [47], for example, and expressed by Equation (8):

*A* = *ε* × *c* × *l* (8)

where the *A* parameter represented the absorbance of a solution, the descriptor *ε* was a molar absorption coefficient (in the L/mol/cm units) and *l* was the path length (in the cm units).

### 3.5. Biological Assays

#### 3.5.1. _In Vitro_ Antimycobaterial Evaluation

*Mycobacterial strains*. The _in vitro_ activity of compounds **8a**–**h** was inspected against *Mycobacterium tuberculosis* CNCTC My 331/88 (identical with H_37_R_v_ and ATCC 2794, respectively; dilution of the strain was 10^−3^ M), *M. kansasii* CNCTC My 235/80 (identical with ATCC 12478; 10^−4^ M), the *M. kansasii* 6 509/96 clinical isolate (10^−4^ M) and *M. avium* CNCTC My 330/80 (identical with ATCC 25291; 10^−5^ M), respectively, in the Laboratory for Mycobacterial Diagnosis and Tuberculosis (Institute of Public Health in Ostrava, Czech Republic). These strains were purchased from the National Reference Laboratory – Czech National Collection of Type Cultures (CNCTC; The National Institute of Public Health, Prague, Czech Republic), excluding *M. kansasii* 6 509/96, which was clinically isolated because the INH-resistant *M. kansasii* strains have not been found in Czech Republic or Slovak Republic yet.

*M. avium intracellulare* ATCC 13950 as well as the *M. kansasii* CIT11/06, *M. avium* subsp. *paratuberculosis* CIT03 and *M. avium hominissuis* CIT10/08 clinical isolates, respectively, were obtained from the Department of Biological Sciences (Cork Institute of Technology, Bishopstown, Cork, Ireland).

*Standard drugs*. The isoniazid (INH), ethambutol (EMB), ofloxacin (OFLX), ciprofloxacin (CPX) and pyrazinamide (PZA) reference drugs were purchased from Sigma-Aldrich (Darmstadt, Germany), showing the purity of analytical grade.

*Determination of a minimum inhibitory concentration (MIC) against M. tuberculosis CNCTC My 331/88, M. kansasii CNCTC My 235/80, M. kansasii 6 509/96 and M. avium CNCTC My 330/80*. Efficiency of the compounds **8a**–**h** and standard drugs against given mycobacteria were determined in the Šula semisynthetic medium (Sevac, Prague, Czech Republic) by a dilution-micromethod [48,76].

In a brief, each mycobacterial strain was simultaneously inoculated into Petri plates containing a Löwenstein-Jensen medium for sterility control and growth of inoculum. All inspected compounds were added to the medium as solutions in dimethyl sulfoxide (DMSO; Sigma-Aldrich, Irvine, UK). In the assays, following concentrations of the solutions were used: 1000, 500, 250, 125, 62.5, 32, 16, 8, 4, 2, 1, 0.5, 0.25 and 0.125 μM, respectively. The inoculated plates kept in microtone bags were incubated at 37 °C. Particular reading was carried out on a stand with a bottom magnifying mirror, macroscopically, with the use of a magnifying glass. The growth in the plates was evaluated after 7, 14 and 21 days (*M. kansasii* CNCTC My 235/80, *M. kansasii* 6 509/96) and after 14 and 21 days (*M. tuberculosis* CNCTC My 331/88, *M. avium* CNCTC My 330/80), respectively [48,76].

The value of a minimum inhibitory concentration (*MIC*) was the lowest concentration (on the above concentration scale) of a tested compound, which inhibited growth of the mycobacteria [48,76]. The evaluation was repeated three times and the *MIC* values, reported in Table 4 in the μM units, were the same.

*Determination of a minimum inhibitory concentration (MIC) against M. avium intracellulare ATCC 13950, M. kansasii CIT11/06, M. avium subsp. paratuberculosis CIT03 and M. avium hominissuis CIT10/08*. Those mycobacterial strains were grown in a Middlebrook broth (MB), supplemented with Oleic-Albumin-Dextrose-Catalase Supplement (OADC; Becton Dickinson, Oxford, UK). Identification of isolates was performed using biochemical and molecular protocols.

At log phase growth, a culture sample (10 mL) was centrifuged at 15,000 rpm/20 min using a bench top centrifuge Model CR 4-12 (Jouan Inc., London, UK). Following removal of a supernatant, the pellet was washed in a fresh Middlebrook 7H9GC broth (Difco, Detroit, MI, USA), and re-suspended in a fresh supplemented MB (10 mL). Turbidity was adjusted to match the McFarland standard No. 1 containing 3 × 10^8^ Colony Forming Units (CFU) with the MB broth. Further 1:20 dilution of a culture was performed in the broth.

Susceptibility of given mycobacterial strains was investigated in a 96-well plate format. In the experiments, sterile deionised water (300 μL) was added to all outer-perimeter wells of plates to minimize evaporation of a medium in the test wells during the incubation process. Each tested compound (100 μL) was incubated with each of mycobacterial species (100 μL). The dilutions of tested compounds were prepared in triplicate, their final concentrations ranged from 500 μg/mL to 15 μg/mL. The screened derivatives were prepared in DMSO (Sigma-Aldrich, London, UK) and dilutions were made in the supplemented MB broth. The plates were sealed with a parafilm and incubated at 37 °C for 7 days. Following the incubation, 10% addition of a water-soluble dye, the alamarBlue reagent (AbD Serotec, Kidlington, UK) was mixed into each well. Absorbance readings at *λ* = 570 nm and 600 nm were taken, initially for a background subtraction and after 24 h re-incubation. The subtraction is necessary for strongly coloured compounds, where the colour may interfere with interpretation of any colour change. For non-interfering compounds, a blue colour in a well was interpreted as absence of growth and pink colour was scored as growth [17,49,50].

The *MIC* value was defined as the lowest concentration of a compound, at which no visible bacterial growth was observed. In other words, the *MIC* was the lowest concentration that prevented a visual colour change from blue to pink. The *MIC* for mentioned mycobacteria was defined as 90% or greater growth reduction (*IC*_90_) compared to a control. The *MIC* (*IC*_90_) parameter has been routinely and widely used in bacterial assays as a standard detection limit according to the Clinical and Laboratory Standards Institute [49,50].

Clinically used antimycobacterial drugs INH, CPX and PZA, respectively, were applied as standards. The observed *MIC* values were listed in Table 5 in the μM units.

#### 3.5.2. _In Vitro_ Antiproliferative (Cytotoxicity) Screening

Human monocytic leukemia THP-1 cells were obtained from the European Collection of Cell Cultures (ECACC; Salisbury, UK; Methods of characterization: DNA Fingerprinting (Multilocus probes) and isoenzyme analysis). The cells were routinely cultured in the RPMI 1640 medium (Lonza, Verviers, Belgium) supplemented with 10% fetal bovine serum (Sigma-Aldrich, Darmstadt, Germany), 2% *L*-glutamine, 1% penicillin and streptomycin (Lonza, Verviers, Belgium) at 37 °C with 5% CO_2_. The cells were passaged at approximately 1-week intervals and were routinely tested for absence of mycoplasma by a Hoechst 33258 staining method.

The tested compounds **8a**–**h** were dissolved in DMSO (Sigma-Aldrich, Darmstadt, Germany) and added in five increasing concentrations to the cell suspension in the culture medium. The maximum concentration of DMSO in the assays never exceeded 0.1%. Subsequently, the cells were incubated for 24 h at 37 °C with 5% CO_2_ at various compounds´ concentrations varying from 0.37 μM to 30 μM in the RPMI 1640 medium.

Cell toxicity was determined using the Cytotoxicity Detection KitPLUS Lactate Dehydrogenase (LDH) assay kit (Roche Diagnostics, Mannheim, Germany) and used according to manufacturer’s instructions. For the LDH assays, the cells were seeded into 96-well plates (5 × 10^4^ cells/well in 100 μL culture medium) in triplicate in the serum-free RPMI 1640 medium, and measurements at *λ* = 492 nm (Synergy 2 Multi-Mode Microplate Reader; BioTek, Winooski, VT, USA) were taken 24 h after treatment with the tested compounds [77,78].

Median lethal dose values, *LD*_50_, were deduced through the construction of a dose-response curve. All values were evaluated using the GraphPad Prism 5.00 software (GraphPad Software, San Diego, CA, USA).

### 3.6. Calculations and Statistical Analyses

Regression equations and statistical characteristics were calculated and visualized by the Origin Pro 9.0.0 software (OriginLab Corporation, Northampton, MA, USA).

In a current research, those statistical parameters were calculated: Residual sum of squares (*RSS*), correlation coefficient (*R*), adjusted coefficient of determination (*Adj. R*^2^), root mean squared error (standard deviation; *RMSE*) and norm of residuals (*NoR*), respectively. The study provided Analysis of Variance (ANOVA) outputs as well, i.e., Fisher´s *F*-test (Fisher´s significance ratio; *F*) and probability of obtaining the *F* Ratio (*Prob* > *F*).

The *RSS* descriptor was used to measure amount of variance in a data set that was not explained by the regression model. The *RSS* parameter was a measure of amount of error remaining between the regression function and data set. Relatively smaller *RSS* explained greater amount of data [79,80].

The *R* parameter was based on a method of covariance. The coefficient was used to measure the strength of a relationship between two (continuous) variables. The *Adj. R*^2^ value penalized the *R*^2^ data for addition of regressors, which did not contribute to explanatory power of a model. Indeed, the *Adj. R*^2^ value was never larger than the *R*^2^ one; it would be decreased by the adding of other regressors, and might be even negative for poorly fitting models [81,82,83].

The *RMSE* was an unambiguous indicator of error for numerical predictions. The parameter provided standard deviation of a model prediction error. Relatively smaller value indicated better model performance [84]. The *NoR* parameter was the square root of *RMSE* and was used as measure for goodness of fit when comparing different fits [85].

The *F* value, as a parameter obtained by the ANOVA, could determine whether the means of three or more groups were different. The ANOVA approach tested an effect of a categorical predictor variable on a continuous dependent variable and used the *F*-test to statistically test equality of means [79].

The *Prob > F* data provided a *p*-value for the test and measured probability of obtaining the *F* Ratio as large as what was observed, given that all parameters except the intercept were zero [81,82].

Indication of significance level of the *F* Ratio by stars was as follows: one star, i.e., statistically significant relationship defined by the *Prob > F* value in the range from 0.0100 to <0.0500; two stars, i.e., statistically very significant model defined by the *Prob > F* parameter in the interval from 0.0010 to <0.0100; and three stars, i.e., statistically extremely significant relationship with the *Prob > F* value in the interval from 0 to <0.0010. Finally, a statistically insignificant model was connected with the *Prob > F* parameter ≥ 0.0500 [82].

## 5. Conclusions

In summary, original *N*-arylpiperazines **8a**–**h** were prepared by multistep procedures and characterized by spectral values (^1^H-NMR, ^13^C-NMR, IR and ESI-MS) and elemental analyses (% C, H, N). The compounds contained a flexible 3-/4-alkoxycarbonylamino group (alkoxy = methoxy to butoxy), 2-hydroxyethane-1,2-diyl connecting chain and 4-(2-fluorophenyl)piperazin-1-yl moiety (Table 1). These fragments have been separately found in a chemical structure of various compounds with a notable _in vitro_ efficiency against some tuberculous strains of mycobacteria.

Lipohydrophilic properties of the molecules **8a**–**h** were preliminary evaluated by the RP-TLC using silica gel plates impregnated with 1% silicone oil in heptane. Calculated *R*_M_ values of 3-alkoxycarbonylamino substituent-containing compounds (**8a**–**d**) ranged from −0.55 (**8a**) to 0.01 (**8d**), the 4-substituted ones (**8e**–**h**) showed higher *R*_M_ parameters varying from −0.02 (**8e**) to 0.63 (**8h**).

Linearly extrapolated logarithms of retention factors corresponding to 100% water as a mobile phase, log *k*_w_ values (RP-HPLC), characterized the lipohydrophilic properties of the molecules **8a**–**h** (Table 2) more reliably and precisely than any arbitrary selected isocratic log *k* parameters. These log *k*_w_ values were in accordance with elution order and hydrophobicity of **8a**–**h** and ranged from 2.113 (**8e**) to 2.930 (**8h**). The derivatives **8a**–**c** showed higher log *k*_w_ values (2.430–2.796) than the molecules **8e**–**g** (2.113–2.600). The compounds containing the longest side chain, i.e., 1-(2-{3-[(butoxycarbonyl)amino]phenyl}-2-hydroxyethyl)-4-(2-fluorophenyl)piperazin-1-ium chloride (**8d**) and 1-(2-{4-[(butoxycarbonyl)amino]phenyl}-2-hydroxyethyl)-4-(2-fluorophenyl)-piperazin-1-ium chloride (**8h**) were found to be the most lipophilic, as proven by their log *k*_w_ of 2.796 (**8d**) and 2.930 (**8h**), respectively (Table 2).

Regarding the extrapolation calculations, the slopes *S* of regression lines varied from 2.7386 (**8e**) to 3.3441 (**8h**; Table 2). The *S* parameter was related to a specific hydrophobic surface of a particular compound and could be used as the alternative measure of its lipophilicity.

Electronic properties of the inspected compounds **8a**–**h** were characterized by logarithms of molar absorption coefficients (log ε) of their methanolic solutions (*c* = 3.0 × 10^−5^ M) investigated in the UV/Vis region of a spectrum.

The solutions showed three absorption maxima in a near ultraviolet (quarz) region of the electromagnetic spectrum, e.g., *λ*_1_ = 208–210 nm, *λ*_2(Ch-T)_ = 238–240 nm and *λ*_3_ = 274–276 nm, respectively (Table 3). The log *ε*_2(Ch-T)_ parameters of the compounds **8a**–**d** observed at a charge-transfer absorption maximum *λ*_2(Ch-T)_ were found in a narrow interval from 4.30 (**8a**) to 4.37 (**8c**). The methanolic solutions of **8e**–**h** were characterized by higher log ε_2(Ch-T)_ values than the ones of **8a**–**d** and these parameters ranged from 4.42 (**8h**) to 4.67 (**8e**; Table 3). In addition, elongation of a 4-side chain led to lower log ε values related to all observed absorption maxima (Table 3).

The racemic compounds **8a**–**h** were _in vitro_ screened against *Mycobacterium tuberculosis* CNCTC My 331/88 (identical with H_37_R_v_ and ATCC 2794), *M. kansasii* CNCTC My 235/80 (identical with ATCC 12478), a *M. kansasii* 6 509/96 clinical isolate, *M. avium* CNCTC My 330/80 (identical with ATCC 25291) and *M. avium intracellulare* ATCC 13950 as well as against *M. kansasii* CIT11/06, *M. avium* subsp. *paratuberculosis* CIT03 and *M. avium hominissuis* CIT10/08 clinical isolates, respectively (Table 4 and Table 5). The biological evaluation revealed the most promising potential of the **8a**–**h** set against *M. tuberculosis*, *M. kansasii* My 235/80 and *M. kansasii* 6 509/96, respectively.

A position and length of a side chain notably affected the activity of presently investigated *N*-arylpiperazine derivatives against *M. tuberculosis* CNCTC My 331/88. The 4-positional isomers were more effective, with the *MIC* values ranging from 8 μM (**8h**) to 125 μM (**8e**), than the 3-positional ones, which possessed the *MIC*s from 32 μM (**8d**) to 250 μM (**8a**). Among all _in vitro_ screened molecules, the INH standard was found to be the most active with the *MIC* = 0.5 μM (14-d/21-d; Table 4).

The compounds **8a**–**d** were more efficient against *M. kansasii* My 235/80 and *M. kansasii* 6 509/96 than the derivatives **8e**–**h**. The most active molecule against given mycobacteria was **8d** with the *MIC* = 16 μM and 62.5 μM, respectively, depending on a particular strain and also on the number of days of incubation. Increase in length of the side chain resulted in lower *MIC* values of **8a**–**d** against these mycobacterial strains. The observed *MIC* parameters were, however, higher compared to the ones related to EMB with the *MIC* = 1 μM and 2 μM (14-d/21-d), or OFLX, which showed the *MIC* = 0.5 μM and 1 μM, respectively (14-d/21-d; Table 4).

The activity of **8a**–**h** against a non-tuberculous INH-resistant *M. avium* CNCTC My 330/80 was apparently dependent on the position of an alkoxycarbonylamino chain. Its presence in the 3-position (**8a**–**d**) led to the *MIC* values varying from 62.5 μM (**8d**) to 500 μM (**8a**; 14-d/21-d). However, a potential of the 4-substituent-containing derivatives (**8e**–**h**) to fight given *mycobacterium* was insufficient (*MIC* > 250 μM; Table 4).

The efficiency of the most active substance **8d** (*MIC* = 62.5 μM; 14-d/21-d) against *M. avium* CNCTC My 330/80 was comparable to the effectiveness of OFLX (*MIC* = 32 μM and 62.5 μM, respectively; 14-d/21-d); reference EMB drug was moderately more active (*MIC* = 16 μM; 14-d/21-d). Elongation of a 3-side chain led to more promising compounds (Table 4).

Regarding current *SAR* studies, lipophilic properties represented by extrapolated log *k*_w_ values seemed to be considerably more important for the _in vitro_ activity of the **8a**–**d** set against *M. tuberculosis* and *M. kansasii* 6 509/96 compared to the lipophilic features of the compounds **8e**–**h**.

The log *ε*_2(Ch-T)_ values (Table 3) observed at the charge-transfer absorption maximum *λ*_2(Ch-T)_ were also taken into a special consideration, because they could be the most sensitive to the differences in a position and electronic properties of the alkoxycarbonylamino substituent.

A relationship between the log *ε*_2__(__Ch-T)_ parameters and log (1/*MIC* [M]) values connected with the _in vitro_ screening of **8a**–**h** against *M. tuberculosis* My 331/88 (14-d) provided a bilinear course. Maximal efficiency of these compounds could be observed if their log ε_2__(__Ch-T)_ values were approximately 4.43 (Figure 4).

If attention was paid to the *M. kansasii* My 235/80 and *M. kansasii* 6 509/96 strains, no significant relationships between the _in vitro_ activity of the compounds **8a**–**h** and their electronic features were observed.

Favorable cytotoxicity profiles of the molecules **8a**–**h** were proved by the *LD*_50_ values > 30 μM, which were estimated on a human monocytic leukemia THP-1 cell line. Moreover, the least lipophilic methoxy group-containing derivatives **8e** (log *k*_w_ = 2.113) and **8a** (log *k*_w_ = 2.430) increased proliferation of the THP-1 cells in 24 h when compared to a control.

Overall, the results of current _in vitro_ biological evaluation and initial *SAR* investigations of the molecules **8a**–**h** considered them very promising candidates for further structural optimization, which could lead to even more effective antimycobacterials. Regarding these findings, the authors of the study were inspired and encouraged to synthesize enantiomerically pure compounds and explore their _in vitro_ efficiency, especially against *M*. *tuberculosis* CNCTC My 331/88, *M. kansasii* My 235/80 or *M. kansasii* 6 509/96, in further phases of the research programme 

## Figures and Tables

**Table 1:** Chemical structure of the compounds **8a**–**h**, their lipophilicity indices *R*_M_ (RP-TLC) and log *k* (RP-HPLC) as well as retention times *t*_r_ (RP-HPLC) estimated in the mobile phases consisted of a various volume ratio (*v*/*v*) of a methanol (MeOH) organic modifier and water.

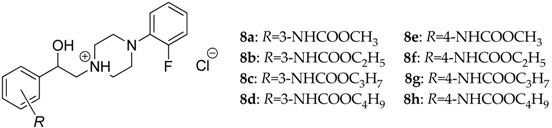									
Comp.	^1^ *R*_M_	Mobile phase MeOH/water (*v*/*v*)							
		60:40		70:30		80:20		85:15	
		*t*_r_ (min)	log *k*	*t*_r_ (min)	log *k*	*t*_r_ (min)	log *k*	*t*_r_ (min)	log *k*
**8a**	−0.55	6.593	0.612	3.587	0.248	2.493	−0.034	2.200	−0.156
**8b**	−0.35	7.707	0.695	4.893	0.444	2.907	0.095	2.433	−0.056
**8c**	−0.16	8.933	0.771	5.907	0.552	3.193	0.166	2.620	0.010
**8d**	0.01	10.021	0.829	7.820	0.702	3.586	0.245	2.830	0.074
**8e**	−0.02	5.307	0.491	3.180	0.163	2.360	−0.085	2.120	−0.196
**8f**	0.19	7.153	0.656	3.953	0.312	2.620	0.010	2.275	−0.121
**8g**	0.39	8.280	0.732	5.320	0.492	3.033	0.128	2.533	−0.020
**8h**	0.63	11.035	0.881	7.287	0.665	3.543	0.240	2.814	0.069

^1^ *R*_M_, Lipophilicity index (RP-TLC). Silica gel plates (stationary phases) were impregnated by 1% silicone oil in heptane.

**Table 2:** Extrapolated log *k*_w_ values (RP-HPLC) of the analyzed molecules **8a**–**h** and statistical descriptors (*RSS*, *R*, *Adj. R*^2^, *RMSE*, *NoR*, *F* and *Prob > F*), which characterized a linear relationship between the log *k* and *ϕ*_M_ values for a particular compound. The *ϕ*_M_ parameter was a volume fraction of MeOH in the isocratic elution RP-HPLC.

Comp.	log *k*_w_	^1^ *S*	^2^ *RSS*	^3^ *R*	^4^ *Adj. R*^2^	^5^ *RMSE*	^6^ *NoR*	^7^ *F*	^8^ *Prob* > *F*
**8a**	2.430	3.0678	0.0023	0.9967	0.9902	0.0337	0.0477	305.70	0.0033 **
**8b**	2.546	3.0529	0.0017	0.9975	0.9925	0.0294	0.0415	398.96	0.0025 **
**8c**	2.679	3.1244	0.0051	0.9930	0.9791	0.0504	0.0713	141.72	0.0070 **
**8d**	2.796	3.1641	0.0208	0.9730	0.9201	0.1019	0.1441	35.58	0.0270 *
**8e**	2.113	2.7386	0.0019	0.9965	0.9896	0.0311	0.0440	285.13	0.0035 **
**8f**	2.512	3.1156	0.0009	0.9988	0.9964	0.0208	0.0294	826.63	0.0012 **
**8g**	2.600	3.0739	0.0027	0.9962	0.9885	0.0367	0.0519	259.09	0.0038 **
**8h**	2.930	3.3441	0.0081	0.9903	0.9710	0.0637	0.0901	101.50	0.0097 **

^1^ *S*, Slope; ^2^ *RSS*, residual sum of squares; ^3^ *R*, correlation coefficient; ^4^ *Adj. R*^2^, adjusted coefficient of determination; ^5^ *RMSE*, root mean squared error (standard deviation); ^6^ *NoR*, norm of residuals; ^7^ *F*, Fisher´s significance ratio (Fisher´s *F*-test); ^8^ *Prob > F*, probability of obtaining the *F* Ratio (significance of a whole model). Indication of a significance level of the *F* Ratio was as follows: * (one star), significant; ** (two stars), very significant.

**Table 3:** Wavelengths of the observed absorption maxima (*λ*_1_, *λ*_2(Ch-T)_ and *λ*_3_) and logarithms of the molar absorption coefficients (log *ε*) of compounds´ methanolic solutions (*c* = 3.0 × 10^−5^ M), which were investigated in the UV/Vis region of a spectrum.

Comp.	*λ*_1_ (nm)	log *ε*_1_	*λ*_2(Ch-T)_ (nm)	^1^ log *ε*_2(__Ch-T__)_	*λ*_3_ (nm)	log *ε*_3_
**8a**	210	4.30	238	4.30	276	3.45
**8b**	210	4.31	238	4.33	276	3.40
**8c**	210	4.30	238	4.37	276	3.42
**8d**	210	4.31	238	4.32	276	3.49
**8e**	210	4.61	240	4.67	274	3.67
**8f**	208	4.59	240	4.60	274	3.60
**8g**	210	4.47	240	4.54	274	3.52
**8h**	208	4.34	240	4.42	274	3.42

^1^ log *ε*_2(Ch-T)_, Logarithms of molar absorption coefficients observed at the charge-transfer absorption maximum *λ*_2(Ch-T)_ = 238–240 nm.

**Table 4:** The _in vitro_ activity (the *MIC* values were expressed in the μM units) of currently screened compounds **8a**–**h** and reference drugs isoniazid (INH), ethambutol (EMB) and ofloxacin (OFLX) against *M. tuberculosis* My 331/88 (*M. tuberculosis* H_37_R_v_; *MT* My 331/88), *M. kansasii* My 235/80 (*MK* My 235/80), *M. kansasii* 6 509/96 (*MK* 6 509/96) and *M. avium* My 330/88 (*MA* My 330/88), respectively.

Comp.	*MIC* (μM)									
	*MT* My 331/88		*MK* My 235/80			*MK* 6 509/96			*MA* My 330/88	
	^1^ 14 d	^2^ 21 d	^3^ 7 d	14 d	21 d	7 d	14 d	21 d	14 d	21 d
**8a**	250	250	125	500	1000	125	500	500	500	500
**8b**	125	125	62.5	250	250	62.5	250	250	250	250
**8c**	62.5	62.5	62.5	125	125	**32**	125	125	125	250
**8d**	**32**	**32**	**32**	62.5	62.5	**16**	**32**	62.5	62.5	62.5
**8e**	125	125	125	500	500	125	500	500	250	500
**8f**	**32**	62.5	125	>250	>250	62.5	>125	>125	>250	>250
**8g**	**16**	**16**	125	>250	>250	125	>250	>250	>250	>250
**8h**	**8**	**8**	62.5	>125	>125	125	>250	>250	>250	250
**INH**	0.5	0.5	>250	>250	>250	4	8	8	>250	>250
**EMB**	1	2	1	2	2	1	2	2	16	16
**OFLX**	1	2	0.5	1	1	0.5	0.5	1	32	62.5

**^1^** 14 d, 14-Day cultivation; ^2^ 21 d, 21-day cultivation; ^3^ 7d, 7-day cultivation. The _in vitro_ activities (*MIC* values) of the most promising *N*-arylpiperazines were highlighted by a bold font style in gray.

**Table 5:** The _in vitro_ activity (the *MIC* values were expressed in the μM units) of the inspected compounds **8a**–**h** and reference drugs isoniazid (INH), ciprofloxacin (CPX) and pyrazinamide (PZA) against *M. kansasii* CIT11/06 (*MK* CIT11/06), *M. avium* subsp. *paratuberculosis* CIT03 (*MAP* CIT03), *M. avium intracellulare* ATCC 13950 (*MAI* ATCC 13950) and *M. avium hominissuis* CIT10/08 (*MAH* CIT10/08), respectively.

Comp.	*MIC* (μM)			
	*MK*	*MAP*	*MAI*	*MAH*
	CIT11/06	CIT03	ATCC 13950	CIT10/08
**8a**	>610	>610	>610	>610
**8b**	295	>590	>590	>590
**8c**	>571	>571	>571	>571
**8d**	>553	> 53	>553	>553
**8e**	>610	>610	>610	>610
**8f**	295	>590	>590	>590
**8g**	>571	>571	>71	>571
**8h**	>553	>553	>553	>553
**INH**	>1823	>1823	>1823	>1823
**CPX**	>91	181	181	181
**PZA**	>2031	>2031	>2031	487

## Supplementary Materials

The supplementary materials are available online at www.mdpi.com/link.

## Abbreviations

The following abbreviations are used in this manuscript:

7-d	7-Day incubation
14-d/21-d	14-/21-Day incubation
*ϕ*_M_	Volume fraction of a mobile phase modifier (RP-HPLC)
CPX	Ciprofloxacin
*Adj. R*^2^	Adjusted coefficient of determination (statistical analysis)
DMSO	Dimethyl sulfoxide
EMB	Ethambutol
*F*	Fisher´s *F*-test (Fisher´s significance ratio; statistical analysis)
INH	Isoniazid
*k*	Capacity (retention) factor (RP-HPLC)
log *k*_w_	Lipophilicity index; values extrapolated from intercepts of a linear relationship between the logarithm of retention factor *k* (log *k*) and volume fraction of a mobile phase modifier (*ϕ*_M_; RP-HPLC)
MDR	Multi-drug resistant
MeOH	Methanol
*MA*	*Mycobacterium avium*
*MIC*	Minimum inhibitory concentration (in the μΜ units)
*MK*	*Mycobacterium kansasii*
Mp/mp	Melting point
*MT*	*Mycobacterium tuberculosis*
*NoR*	Norm of residuals (statistical analysis)
OFLX	Ofloxacin
*Prob > F*	Probability of obtaining the *F* Ratio (statistical analysis)
PZA	Pyrazinamide
*R*_M_	Lipophilicity index (RP-TLC)
*RMSE*	Root mean squared error (statistical analysis)
*RSS*	Residual sum of squares (statistical analysis)
*S*	Slope (RP-HPLC)
*SAR*	Structure–activity relationship(s)
*t*_r_	Retention time of a compound (RP-HPLC)
