# Supplementary material for: Synthesis and In Vitro Antimycobacterial Activity of Novel N-Arylpiperazines Containing an Ethane-1,2-diyl Connecting Chain
Source: Molecules. 2017 Nov 30;22(12):2100. doi: 10.3390/molecules22122100 (PMC6149664; doi:10.3390/molecules22122100)
Supplement: Supplementary file 1 [file molecules-22-02100-s001.pdf]

Supplementary Material

Synthesis and *In Vitro* Antimycobacterial Activity of Novel *N*-Arylpiperazines Containing an Ethane-1,2-diyl Connecting Chain

Tomáš Goněc [1], Ivan Malík [2,*], Jozef Csöllei [1], Josef Jampílek [2], Jiřina Stolaříková [3], Ivan Solovič [4,5], Peter Mikuš [6], Stanislava Keltošová [7], Peter Kollár [7], Jim O'Mahony [8] and Aidan Coffey [8]

[1] Department of Chemical Drugs, Faculty of Pharmacy, University of Veterinary and Pharmaceutical Sciences in Brno, Palackého 1946/1, Brno CZ-612 42, Czech Republic; gonect@vfu.cz (T.G.), csolleij@vfu.cz (J. Cs.)

[2] Department of Pharmaceutical Chemistry, Faculty of Pharmacy, Comenius University in Bratislava, Odbojárov 10, Bratislava SK-832 32, Slovak Republic; malikivan001@gmail.com (I.M.); josef.jampilek@gmail.com (J.J.)

[3] Laboratory for Mycobacterial Diagnostics and Tuberculosis, Regional Institute of Public Health, Partyzánské náměstí 7, Ostrava CZ-702 00, Czech Republic; Jirina.Stolarikova@zu.cz (J.S.)

[4] Clinic for Tuberculosis and Lung Diseases, National Institute for Tuberculosis, Lung Diseases and Thoracic Surgery, Vyšné Hágy, Vysoké Tatry SK-059 84, Slovak Republic; solovic@hagy.sk (I.S.)

[5] Department of Public Health, Faculty of Health, Catholic University in Ružomberok, Hrabovská cesta 1A, Ružomberok SK-034 01, Slovak Republic; ivan.solovic@ku.sk (I.S.)

[6] Department of Pharmaceutical Analysis and Nuclear Pharmacy, Faculty of Pharmacy, Comenius University in Bratislava, Odbojárov 10, Bratislava SK-832 32, Slovak Republic; mikus@fpharm.uniba.sk (P.M.)

[7] Department of Human Pharmacology and Toxicology, University of Veterinary and Pharmaceutical Sciences in Brno, Palackého 1946/1, Brno CZ-612 42, Czech Republic; kollarp@vfu.cz (P.K.); keltosovas@vfu.cz (S.K.)

[8] Department of Biological Sciences, Cork Institute of Technology, Bishopstown, Cork T12 P928, Ireland; jim.omahony@cit.ie (J.O.M.); aidan.coffey@cit.ie (A.C.)

* Correspondence: malikivan001@gmail.com (I.M.); Tel.: +421-2-501-117-227

Table S1. Observed R_f values and calculated R_M parameters (RP-TLC) of the evaluated compounds **8a–h** using stationary phases (silica gel plates) impregnated with a variously concentrated silicone oil in heptane.

Entry	1%		3%		5%	
	R_f	R_M	R_f	R_M	R_f	R_M
**8a**	0.78	-0.55	0.59	-0.16	0.53	-0.05
**8b**	0.69	-0.35	0.52	-0.03	0.44	0.11
**8c**	0.59	-0.16	0.42	0.42	0.27	0.44
**8d**	0.49	0.01	0.27	0.27	0.19	0.64
**8e**	0.51	-0.02	0.39	0.19	0.36	0.25
**8f**	0.39	0.19	0.33	0.31	0.28	0.41
**8g**	0.29	0.39	0.21	0.58	0.22	0.55
**8h**	0.19	0.63	0.13	0.83	0.13	0.83

Table S2. Observed values of capacity factors *k* (RP-HPLC) of the investigated compounds **8a–h**, which were determined in the methanol (MeOH)/water mobile phases containing a varying volume ratio (*v*/*v*) of the organic modifier.

Entry	*k*; MeOH/water (*v*/*v*)			
	k; 60:40	*k*; 70:30	*k*; 80:20	*k*; 85:15
**8a**	4.091	1.770	0.925	0.699
**8b**	4.951	2.778	1.245	0.879
**8c**	5.898	3.561	1.466	1.023
**8d**	6.738	5.039	1.769	1.185
**8e**	3.098	1.456	0.822	0.637
**8f**	4.524	2.053	1.023	0.757
**8g**	5.394	3.108	1.342	0.956
**8h**	7.598	4.627	1.736	1.173

Table S3. Relationships between the log k_w values and *in vitro* activity (in the log (1/*MIC* [M]) units) of the compounds under the study.

Equation No.	Strain (Days of Cultivation)/Series	Equation	Statistical Descriptors
**Eq. (S1)**	[1] ***MT* (14-d)/ 8a–d**	log (1/*MIC* [M]) = 2.4188 (±0.0431) × log k_w – 2.2686 (±0.1129)	[3] *RSS*=0.0003, [4] *R*=0.9997, [5] *Adj. R*^{2}=0.9991, [6] *RMSE*=0.0119, [7] *NoR*=0.0168, [8] *F*=3143.04, [9] *Prob>F*=0.0003 ***, [10] *n*=4
**Eq. (S2)**	***MT* (14-d)/ 8e–h**	log (1/*MIC* [M]) = 1.4933 (±0.1864) × log k_w + 0.7816 (±0.4762)	*RSS*=0.0235, *R*=0.9849, *Adj. R*^{2}=0.9547, *RMSE*=0.1085, *NoR*=0.1534, *F*=64.20, *Prob>F*=0.0152 **, *n*=4
**Eq. (S3)**	***MT* (21-d)/ 8a–d**	log (1/*MIC* [M]) = 2.4188 (±0.0431) × log k_w – 2.2686 (±0.1129)	*RSS*=0.0003, *R*=0.9997, *Adj. R*^{2}=0.9991, *RMSE*=0.0119, *NoR*=0.0168, *F*=3143.04, *Prob>F*=0.0003 ***, *n*=4
**Eq. (S4)**	***MT* (21-d)/ 8e–h**	log (1/*MIC* [M]) = 1.5162 (±0.4007) × log k_w + 0.6506 (±1.0239)	*RSS*=0.1088, *R*=0.9367, *Adj. R*^{2}=0.8162, *RMSE*=0.2332, *NoR*=0.3298, *F*=14.32, *Prob>F*=0.0633, *n*=4
**Eq. (S5)**	[2] ***MK* (7-d)/ 8a–d**	log (1/*MIC* [M]) = 1.4282 (±0.3673) × log k_w + 0.4700 (±0.9610)	*RSS*=0.0205, *R*=0.9398, *Adj. R*^{2}=0.8248, *RMSE*=0.1011, *NoR*=0.1430, *F*=15.12, *Prob>F*=0.0602, *n*=4

[1] *MT*, *Mycobacterium tuberculosis* My 331/88 (*M. tuberculosis* $H_{37}R_v$); [2] *MT*, *Mycobacterium kansasii* My 235/80; [3] *RSS*, residual sum of squares; [4] *R*, correlation coefficient; [5] *Adj. R*^{2}, adjusted coefficent of determination; [6] *RMSE*, root mean squared error (standard deviation); [7] *NoR*, norm of residuals; [8] *F*, Fisher´s significance ratio (Fisher´s *F*-test); [9] *Prob>F*, probability of obtaining the *F* Ratio (significance of a whole model); [10] *n*, number of cases (points). The indication of a significance level of the *F* Ratio: * (one star), statistically significant; ** (two stars), statistically very significant; *** (three stars), statistically extremely significant. The insignificant relationships were indicated by a red colour.

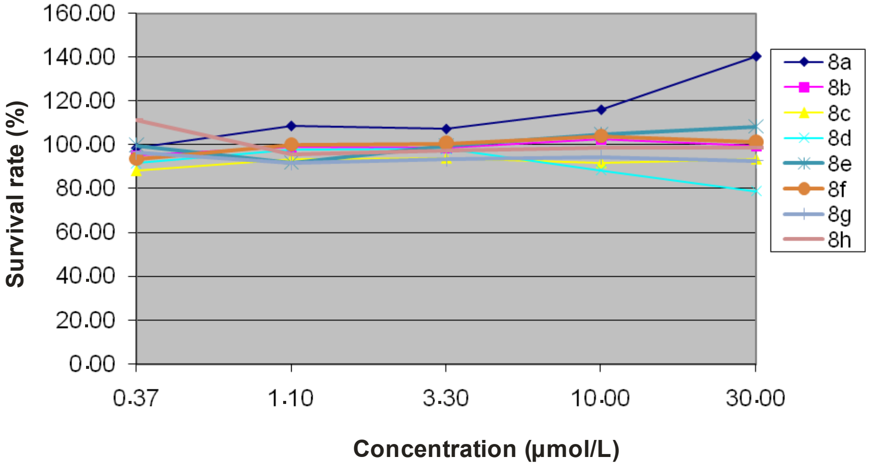

Figure S1. Survival rate curves of the compounds **8a–h** in a THP-1 cell line after the 24 h treatment.
